# Mast cell activation by NGF drives the formation of trauma-induced heterotopic ossification

**DOI:** 10.1172/jci.insight.179759

**Published:** 2024-11-26

**Authors:** Tao Jiang, Xiang Ao, Xin Xiang, Jie Zhang, Jieyi Cai, Jiaming Fu, Wensheng Zhang, Zhenyu Zheng, Jun Chu, Minjun Huang, Zhongmin Zhang, Liang Wang

**Affiliations:** 1 Division of Spine Surgery, Department of Orthopedics, The Third Affiliated Hospital, Southern Medical University, Academy of Orthopedics, Guangdong Province, Guangzhou, China.; 2 Division of Spine Surgery, Department of Orthopedics, Nanfang Hospital, Southern Medical University, Guangzhou, China.; 3 Department of General Medicine, Zhujiang Hospital, Southern Medical University, Guangzhou, China.

**Keywords:** Bone biology, Immunology, Bone disease, Mast cells

## Abstract

Soft tissue trauma can cause immune system disturbance and neuropathological invasion, resulting in heterotopic ossification (HO) due to aberrant chondrogenic differentiation of mesenchymal stem cells (MSCs). However, the molecular mechanisms behind the interaction between the immune and nervous systems in promoting HO pathogenesis are unclear. In this study, we found that mast cell–specific deletion attenuated localized tissue inflammation, with marked inhibition of HO endochondral osteogenesis. Likewise, blockage of nerve growth factor (NGF) receptor, known as tropomyosin receptor kinase A (TrkA), led to similar attenuations in tissue inflammation and HO. Moreover, while NGF/TrkA signaling did not directly affect MSCs chondrogenic differentiation, it modulated mast cell activation in traumatic soft tissue. Mechanistically, lipid A in LPS binding to TrkA enhanced NGF-induced TrkA phosphorylation, synergistically stimulating mast cells to release neurotrophin-3 (NT3), thereby promoting MSC chondrogenic differentiation in situ. Finally, analysis of single-cell datasets and human pathological specimens confirmed the important role of mast cell–mediated neuroinflammation in HO pathogenesis. In conclusion, NGF regulates mast cells in soft tissue trauma and drives HO progression via paracrine NT3. Targeted early inhibition of mast cells holds substantial promise for treating traumatic HO.

## Abstract 2

NGF-activated mast cells induce an aberrant stem cell fate decision via paracrine NT3. Early intervention that disrupts neuroinflammatory signals post-trauma may help treat heterotopic ossification.

## Graphical abstract

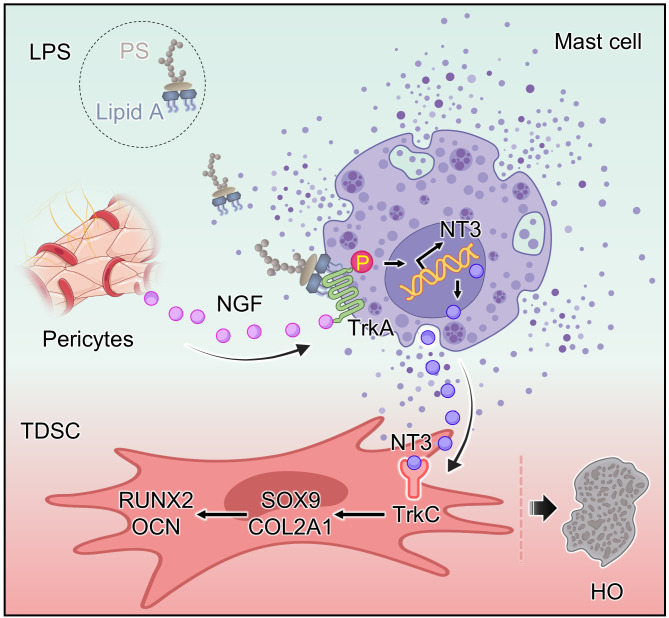

## Introduction

Heterotopic ossification (HO) is a pathological process that abnormally initiates bone tissue formation within soft tissues, including muscles, ligaments, and tendons (1). HO can cause chronic pain, compromised joint function, and subsequent disability, profoundly affecting the quality of life (2). Unfortunately, our limited understanding of the pathogenesis of HO hinders the development of effective therapies (3). Trauma is the most common trigger for the development of HO, primarily due to the inflammation it induces (4). Early-stage inflammation following soft tissue trauma disrupts local stem cell normal repair mechanisms, and thus plays a substantial role in the onset of traumatic HO (5, 6). Patients with nerve injuries often have an intensified inflammatory response and heightened susceptibility to HO, suggesting a link between severe inflammation driven by neurotransmitters and HO development (4, 7, 8). Mast cells act as sentinels in the traumatic microenvironment, bridging neurological and immune functions within it (9). Thus, the neuroinflammatory response mediated by them may contribute to traumatic HO.

Although evidence suggests that mast cells play a pathogenic role in fibrodysplasia ossificans progressiva (FOP; a type of hereditary HO), how they influence traumatic HO remains unclear (10–12). Traumatic HO is a typical endochondral ossification process, with initial chondrogenesis at the trauma site being crucial for its development (13). Recently, a study found that pericyte-derived nerve growth factor (NGF) can bind to its specific receptor, tropomyosin receptor kinase A (TrkA), to induce sensory nerve invasion and promote abnormal osteochondral differentiation of tissue-specific stem cells (14). However, the relationship between mast cells and NGF in the pathogenesis of HO is not yet understood. NGF, as a fundamental neuropeptide, can not only regulate nerve cell growth and survival but it can also act as a chemokine to induce mast cells accumulation (15, 16). Additionally, NGF has been observed to enhance antigen-induced mast cell activation, facilitating the secretion of various regulatory factors in vitro (17–19). Based on these findings, it is important to determine if mast cells are also affected by NGF and accelerate HO progression in posttraumatic soft tissue.

In this study, we sought to examine the crosstalk between NGF and mast cells using a previously described trauma-induced HO model (20, 21). During traumatic HO progression, there was extensive infiltration of mast cells and NGF within the injured soft tissue. Both mast cell–specific deletion and inhibition of TrkA impeded local tissue inflammation, thus abrogating ectopic endochondral osteogenesis. Unexpectedly, tissue-specific stem cells exhibited scarce TrkA but abundant TrkC expression. Furthermore, we found that NGF and lipid A in LPS cobind to TrkA, activating mast cells to secrete neurotrophin-3 (NT3; a ligand of TrkC). Overactive NT3/TrkC signaling induces an aberrant stem cell fate decision. Finally, analyses of mouse single-cell datasets and human pathological specimens confirmed the relevance of mast cells activated by NGF in HO. In summary, NGF activates mast cells to secrete NT3, causing abnormal stem cell differentiation and ultimately leading to HO formation. These insights could provide a clearer understanding of neuroinflammation-associated HO and offer a potential therapeutic target to mitigate traumatic HO progression.

## Results

### Mast cell deletion inhibits pathological endochondral osteogenesis in the posttraumatic mouse model.

**Figure 1:**
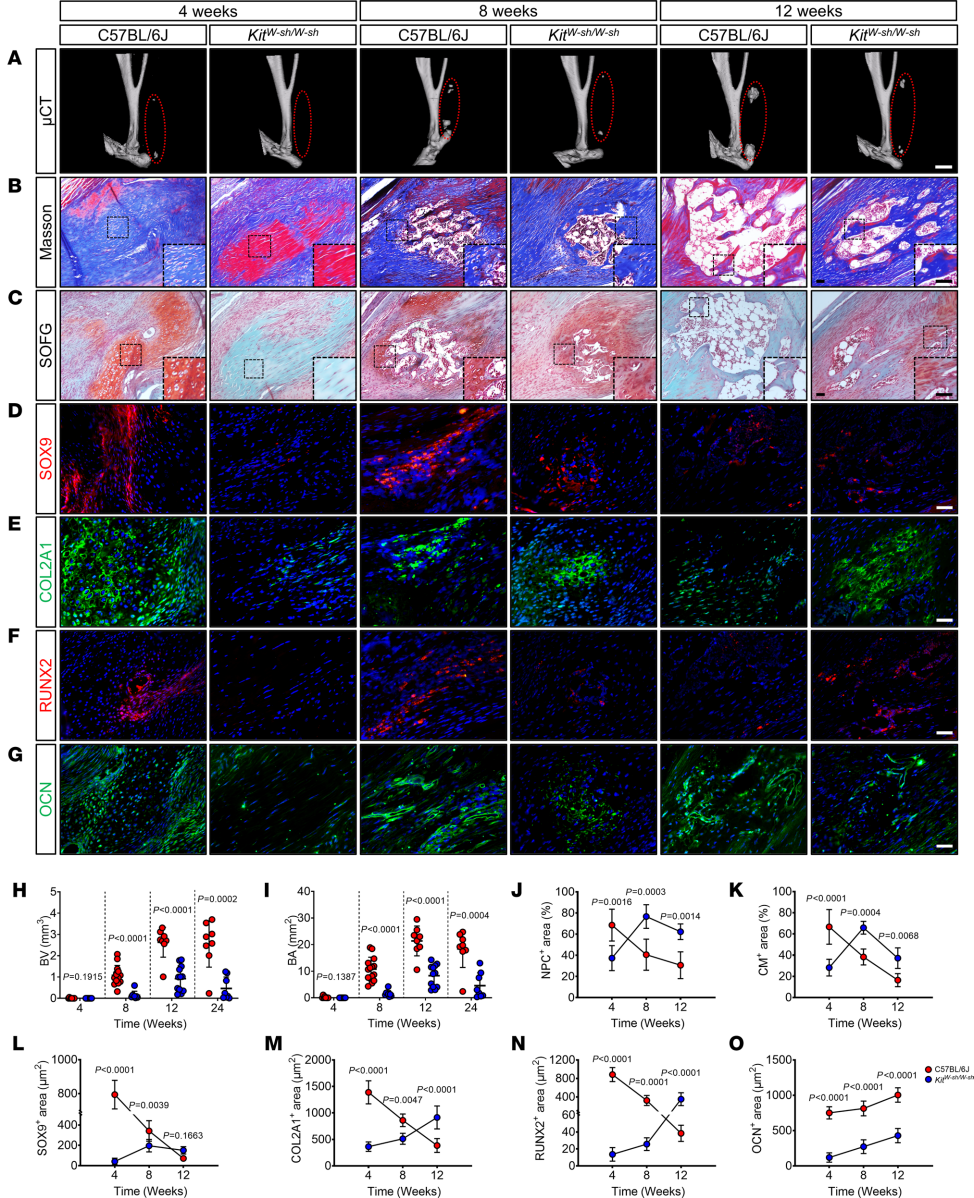
Mast cell deficiency inhibits pathological endochondral osteogenesis. (**A**) Representative μCT 3D modeling images of Achilles tendon (sagittal view) in the indicated groups 4 weeks (cartilaginous phase), 8 weeks (ossification phase), or 12 weeks (osseous phase) after tenotomy. Red dashed ovals represent ectopic bones. Scale bar: 2 mm. (**B** and **C**) Representative images for Masson’s staining (cartilage [blue], heterotopic bone and Achilles tendon [red]) and SOFG staining (cartilage [red], heterotopic bone and Achilles tendon [green]) of injured tendon sections in the indicated groups after tenotomy. Scale bar: 5 μm. (**D**–**G**) Representative IF staining for SOX9 (red), COL2A1 (green), RUNX2 (red), and OCN (green) of injured tendon sections in the indicated groups after tenotomy, with DAPI counterstaining (blue). Scale bar: 5 μm. (**H** and **I**) Representative quantification of **A** and Supplemental Figure 6, showing ectopic bone volume (BV) and bone surface area (BA) in the indicated groups after tenotomy. *n* = at least 8 biological replicates. (**J** and **K**) Representative quantification of **A** and **B**, showing the percentage of neosynthetic pathological collagen (NPC) and cartilage matrix (CM) in injured tendon sections from the indicated groups after tenotomy. *n* = 5 biological replicates. (**L**–**O**) Representative quantification of **D**–**G**, showing the positive areas of SOX9, COL2A1, RUNX2, and OCN in injured tendon sections from the indicated groups after tenotomy. *n* = 5 biological replicates. All data are representative of 2 independent experiments. Data were shown as mean ± SD and compared with 2-tailed unpaired Student’s *t* test (**H** and **I**) or 2-way ANOVA with Šídák’s multiple comparisons test (**J**–**O**).

To explore the role of mast cells in traumatic HO, tenotomy was performed on mast cell–deficient (*Kit^W-sh/W-sh^*) and WT (C57BL/6J) mice. μCT 3D reconstructions disclosed a marked decrease in HO among the *Kit^W-sh/W-sh^* mice compared with the C57BL/6J mice (Figure 1A and Supplemental Figure 6; supplemental material available online with this article; https://doi.org/10.1172/jci.insight.179759DS1). Specifically, 8, 12, and 24 weeks after tenotomy, heterotopic bone volume (BV) and bone surface area (BA) in the injured tendon tissue of *Kit^W-sh/W-sh^* mice were markedly lower (Figure 1, H and I). Masson’s trichrome (Masson’s) staining indicated neosynthetic pathological collagen (NPC), while Safranin O and Fast Green (SOFG) staining revealed cartilage matrix (CM). Both NPC and CM formed during the cartilaginous phase of endochondral ossification (13). At 4 weeks after resection in C57BL/6J mice, NPC and CM were most intense, with the mature bone matrix, indicated by red in Masson’s and green in SOFG staining, progressively intensifying by 8 weeks and becoming dominant by 12 weeks (Figure 1, B, C, J, and K). In *Kit^W-sh/W-sh^* mice, normal tendon tissue remained near the calcaneus at 4 weeks, with markedly lower ectopic osteochondral and mature bone areas at 8 and 12 weeks compared with C57BL/6J mice (Figure 1, B, C, J, and K). While μCT scans showed no statistical difference between groups at 4 weeks after injury, histological staining revealed closer resemblance to normal tendon morphology at the injury site in *Kit^W-sh/W-sh^* mice (Figure 1, A–C).

To further elucidate the pathological characteristics of HO following mast cell–specific deletion, we performed immunofluorescence (IF) staining to assess the expressions of the osteochondral marker proteins SOX9, COL2A1, RUNX2, and OCN in the injured tissues. SOX9 (Figure 1, D and L) and COL2A1 (Figure 1, E and M) expression peaked in C57BL/6J mice at 4 weeks after resection and gradually declined thereafter. In contrast, their expression was lowest at 4 weeks after resection, followed by an increasing trend in *Kit^W-sh/W-sh^* mice. RUNX2 (Figure 1, F and N) expression was highest in C57BL/6J mice at 4 weeks after resection and exhibited a decreasing trend at 8 and 12 weeks. However, RUNX2 expression was nearly absent in *Kit^W-sh/W-sh^* mice 4 weeks after injury and gradually increased at 8 and 12 weeks. In C57BL/6J mice, OCN (Figure 1, G and O) expression was markedly increased at various time points after resection. On the contrary, *Kit^W-sh/W-sh^* mice exhibited low expression at 4 weeks after injury and consistently lower expression than C57BL/6J mice from 8 to 12 weeks. In summary, these results indicate that the deletion of mast cell inhibits ectopic bone maturation, impedes the process of endochondral ossification in HO, and attenuates the extent of HO in posttraumatic tendon tissue.

### Mast cell activation induced by soft tissue trauma involves ectopic osteoblast activity during HO progression.

**Figure 2:**
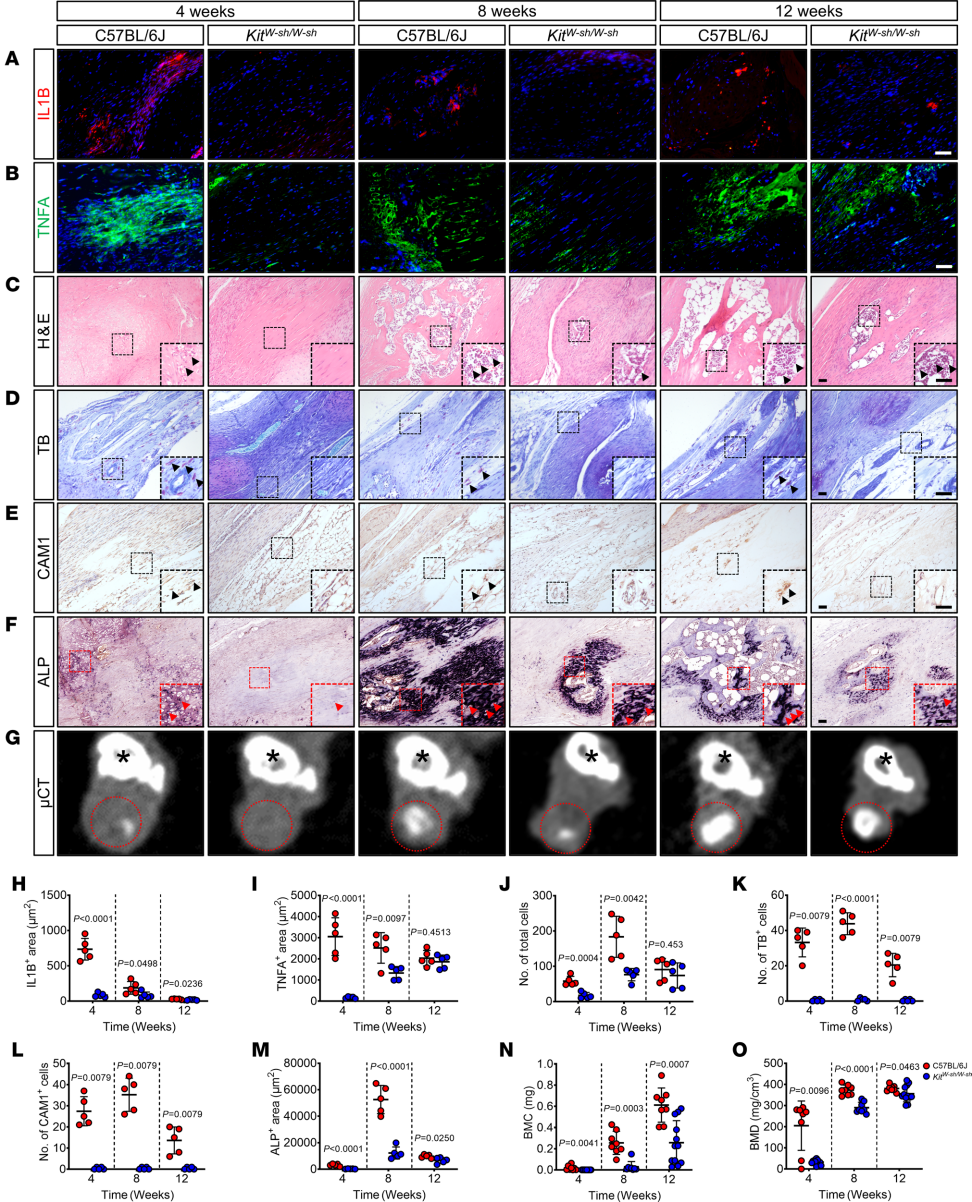
Mast cell activation–induced inflammation correlates with ectopic bone accumulation. (**A** and **B**) Representative IF images for IL1B (red) and TNFA (green) of injured tendon sections in the indicated groups after tenotomy, with DAPI counterstaining (blue). Scale bar: 5 μm. (**C**–**E**) Representative images for H&E staining TB staining (mast cells [violet]), and IHC staining of CAM1 in injured tendon sections from the indicated groups after tenotomy. Scale bar: 5 μm. (**F**) Representative images for alkaline phosphatase (ALP [dark purple]) staining of injured tendon sections in the indicated groups after tenotomy. Scale bar: 5 μm (**G**) Representative CT cross-section of ectopic bone. Black asterisks and red dashed circles represent the tibial and ectopic bone hyperintense images, respectively. (**H** and **I**) Quantification of areas with positive staining for IL1B and TNFA in **A** and **B**. *n* = 5 biological replicates. (**J**–**L**) Quantification of cells with positive staining for H&E (**J**), TB (**K**), and CAM1 (**L**) in **C**–**E**. *n* = 5 biological replicates. (**M**) Quantification of areas with positive staining for ALP in **F**. *n* = 5 biological replicates. (**N** and **O**) Quantification of **G**, showing ectopic bone mineral content (BMC) and mineral density (BMD) in the indicated groups after tenotomy. *n* = at least 8 biological replicates. All data are representative of 2 independent experiments. Data were shown as mean ± SD and compared with 2-tailed unpaired Student’s *t* test.

To ascertain the consequences of mast cell deficiency on the inflammatory response associated with traumatic HO, we examined the expression of IL1B and TNFA in the damaged tissues. A notable decrease in IL-1β expression was observed at 4, 8, and 12 weeks after injury in the *Kit^W-sh/W-sh^* mice (Figure 2, A and H). Likewise, TNFA expression displayed a marked reduction at 4 and 8 weeks but with no statistically significant difference observed at 12 weeks (Figure 2, B and I). Mast cells not only release proinflammatory mediators but also orchestrate the recruitment of peripheral immune cells to the site of inflammation (22). Thus, we further examined the extent of inflammatory cell infiltration within the damaged tissue by H&E staining. Consistent with our predictions, mast cell deletion led to a marked decrease in inflammatory cell infiltration. The peak inflammatory cell count in C57BL/6J mice was recorded at 8 weeks after injury (Figure 2, C and J). However, *Kit^W-sh/W-sh^* mice demonstrated markedly lower inflammatory cell numbers at 4 and 8 weeks compared with C57BL/6J mice (Figure 2, C and J). These observations suggest that mast cell deletion mitigates the local inflammatory response in traumatized tissues.

Tissue toluidine blue (TB) staining showed the presence of mast cells in the traumatized tendon tissue of C57BL/6J mice at 4, 8, and 12 weeks, with a peak cell count observed at 8 weeks after injury (Figure 2, D and K). Mast cell activation status was characterized by IHC staining for the activated mast cell marker chymase (CAM1; encoded by Cam1), revealing similar TB staining results in the injured tendon tissue of C57BL/6J mice (Figure 2, E and L). Conversely, the affected tendon tissue of *Kit^W-sh/W-sh^* mice exhibited no expression of mast cells (Figure 2, D and E). The results of alkaline phosphatase (ALP) staining paralleled the aforementioned staining trend (Figure 2, F and M), which seemed to suggest that the 8-week time point after tissue trauma represented the peak period of HO. Further μCT analysis showed that both the heterotopic bone mineral density (BMD) and bone mineral content (BMC) in *Kit^W-sh/W-sh^* mice were markedly lower than in C57BL/6J mice at corresponding time points after injury (Figure 2, G, N, and O). Collectively, these findings imply that mast cell activation induced by tissue trauma may regulate osteoblast activity, subsequently contributing to abnormal soft tissue ossification.

### Mast cell crosstalk with NGF influences the pathogenesis of traumatic HO.

**Figure 3:**
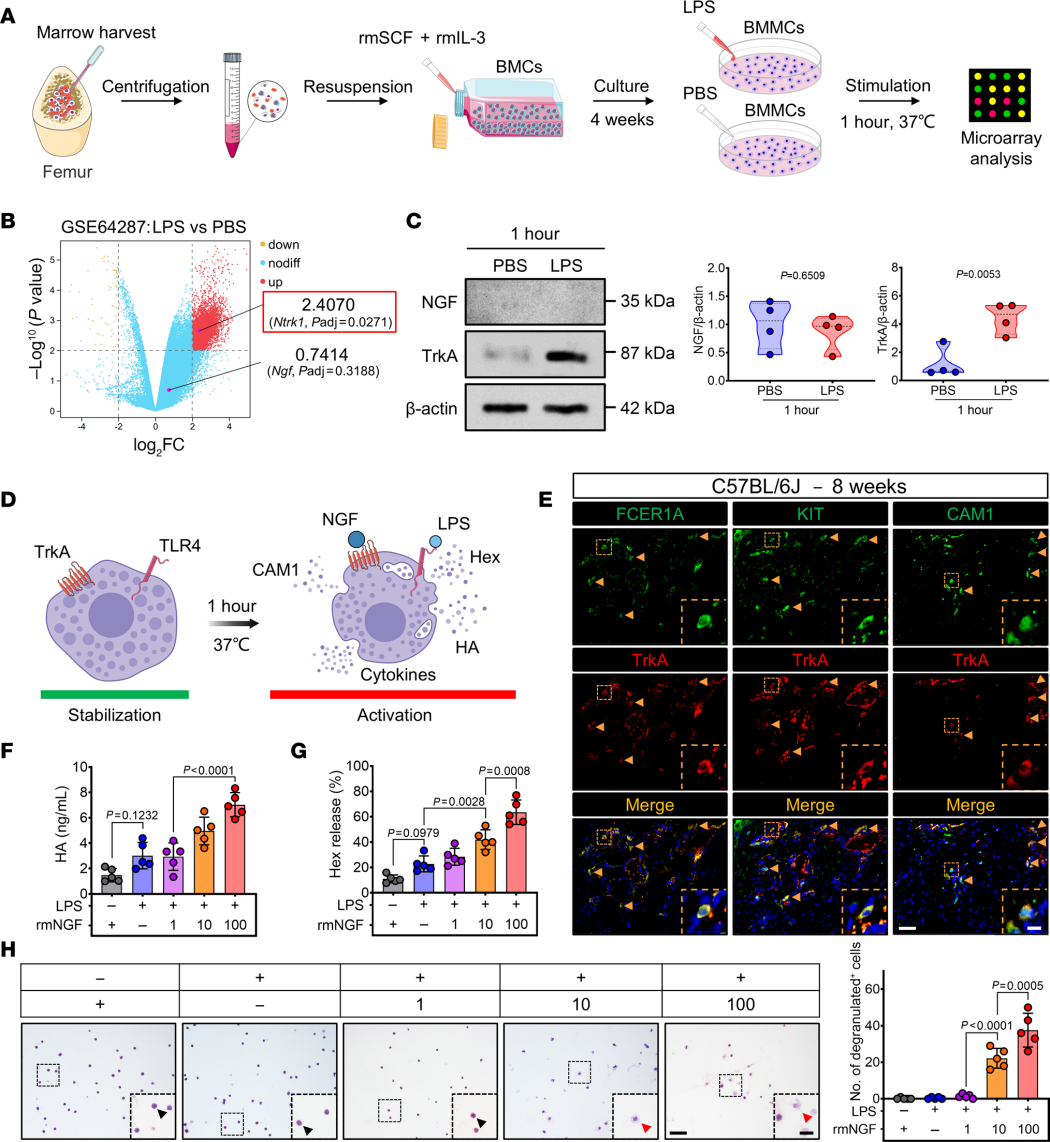
NGF activates mast cell degranulation in traumatized tissue. (**A**) Schematic representation of microarray analysis. (**B**) Volcano plot of upregulated (red) and downregulated (yellow) DEGs in BMMCs treated as shown in **A** from the dataset GSE64287. Expression of the *Ntrk1* (red box) is significantly increased (*P*_adj_ < 0.05, log_2_FC > 2). (**C**) Western blot analysis and densitometric quantification (right) of NGF and TrkA in BMMCs treated as shown in **A**, with β-actin as the loading control. *n* = 4 biological replicates. (**D**) Diagram depicting the degranulation of mast cells coactivated by NGF and LPS. (**E**) Representative IF double-staining images for FCER1A^+^ (green)/TrkA^+^ (red), KIT^+^ (green)/TrkA^+^ (red), and CAM1^+^ (green)/TrkA^+^ (red) cells of injured tendon sections in the indicated group 8 weeks after tenotomy, with DAPI counterstaining (blue). Yellow arrows indicate mast cells. Scale bar: 5 μm (left) and 1 μm (right). (**F** and **G**) BMMCs were separately challenged for 1 hour with rmNGF (100 ng/mL), LPS (100 ng/mL), rmNGF (1 ng/mL) + LPS (100 ng/mL), rmNGF (10 ng/mL) + LPS (100 ng/mL), or rmNGF (100 ng/mL) + LPS (100 ng/mL). (**F**) Histamine (HA) levels and (**G**) β-hexosaminidase (Hex) release ratios were measured by ELISA after the above treatment. *n* = 5 biological replicates. (**H**) Under the conditions previously mentioned (**F** and **G**), TB staining and quantification (right) of degranulated cells among BMMCs were performed. Black arrows indicate resting mast cells, and red arrows indicate degranulated mast cells. Scale bar: 5 μm (left) and 2 μm (right). *n* = 5 biological replicates. Data are representative of 2 independent experiments (**C** and **E**–**H**). Data were shown as mean ± SD and compared with 2-tailed unpaired Student’s *t* test (**C**) or 1-way ANOVA with Tukey’s multiple-comparison test (**F**–**H**).

The inflammatory response induced by bacterial infections is a common trigger for the development of traumatic HO (23, 24). Bacterial-derived LPS is likely to be the key substance leading to the progression of traumatic HO (25). Since mast cells are among the first immune cells to respond to antigens during trauma (9), we initially analyzed the differentially expressed genes (DEGs) in bone marrow–derived mast cells (BMMCs) following LPS treatment in vitro (Figure 3A) (26). Following this, we noted a marked upregulation of *Ntrk1* (the gene encoding TrkA), while *Ngf* (the gene encoding NGF) was not detected in mast cells (Figure 3B). Western blotting further confirmed this finding (Figure 3C). These results suggest that the activation of BMMCs might be regulated by NGF under LPS prestimulation. To test the conjecture, we costimulated BMMCs with LPS at 100 ng/mL and varying concentrations of recombinant murine NGF (rmNGF). Cellular TB staining revealed that treatment with LPS or rmNGF alone did not affect BMMCs (Figure 3H). However, when exposed to a concentration of rmNGF ≥ 10 ng/mL in the presence of LPS (100 ng/mL), BMMCs exhibited cell surface wrinkling and contraction in their morphology (Figure 3H). This characteristic change in cell morphology is known as degranulation (27) and is an indicator of mast cell activation (Figure 3D).

Upon degranulation, mast cells release various inflammatory substances and cytokines (28). To further validate the ability of NGF to induce mast cell degranulation, we collected supernatants from BMMCs treated with both rmNGF and LPS and measured histamine (HA) and β-hexosaminidase (Hex) levels by ELISA. BMMCs exhibited increased release of degranulation markers (HA and Hex) in response to increasing rmNGF concentrations during LPS costimulation (Figure 3, F and G). Furthermore, IF double-staining results revealed coexpression of TrkA with the mast cell–specific markers high-affinity immunoglobulin E receptor alpha chain (FCER1A) and cellular homolog of the feline sarcoma viral oncogene kit (KIT), as well as with CAM1 within the damaged tendon tissue (Figure 3E). Taken together, these findings suggest that, upon LPS stimulation, NGF/TrkA signaling activates mast cells and eventually is involved in the development of traumatic HO.

### NGF regulates the abnormal osteochondral differentiation of tissue-specific stem cells through the activation of mast cells.

**Figure 4:**
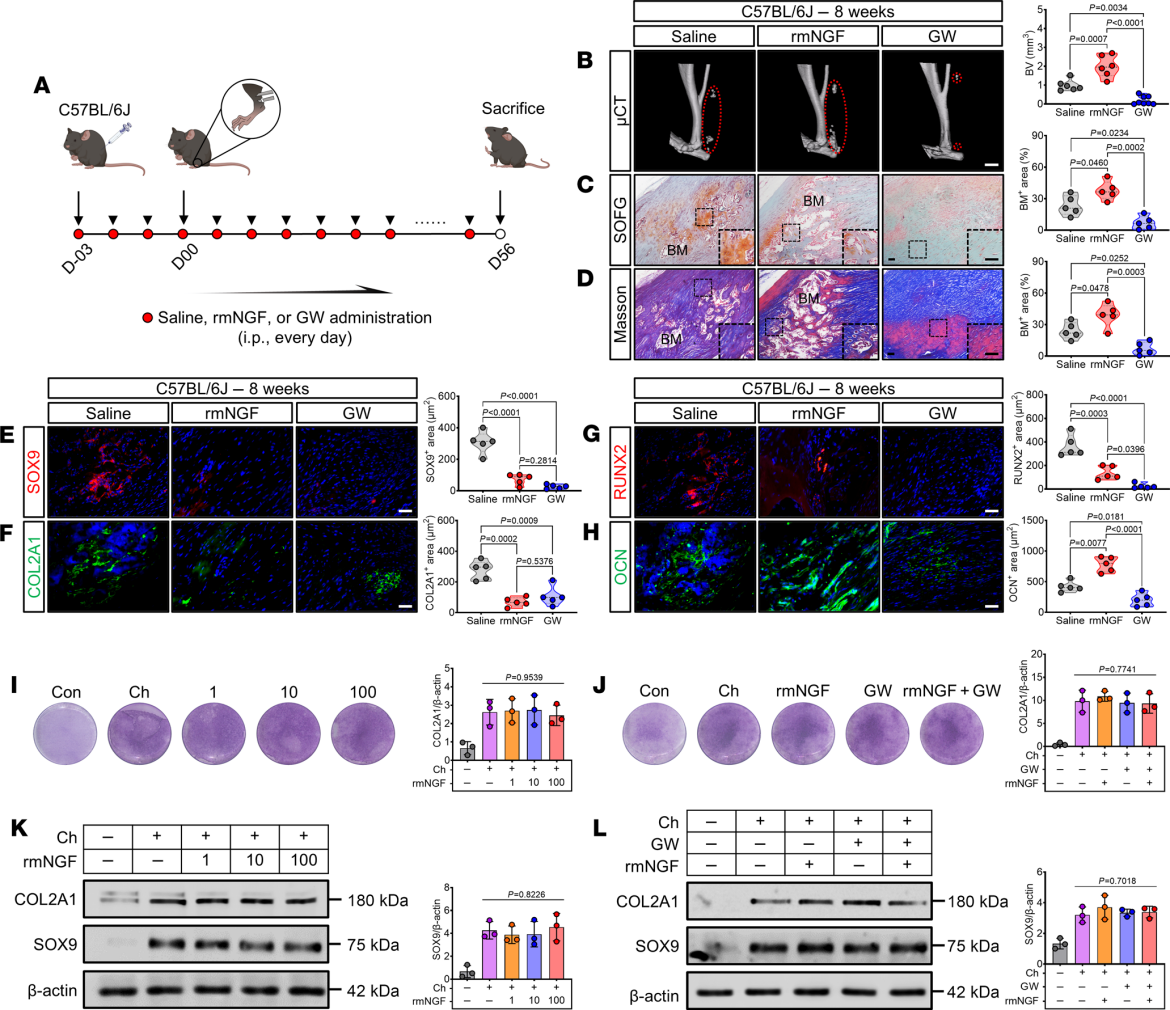
NGF promotes traumatic HO progression without enhancing chondrogenic differentiation of tissue-resident stem cells. (**A**) Schematic representation of saline, rmNGF, or GW administration and traumatic HO induction. (**B**) Representative μCT 3D modeling images and quantification (right) of ectopic BV in Achilles tendon from the indicated groups 8 weeks after tenotomy. Red dashed ovals represent ectopic bones. Scale bar: 2 mm. *n* = at least 6 biological replicates. (**C** and **D**) Representative images for SOFG and Masson’s staining, along with the quantification (right) of bone marrow (BM) areas in injured tendon sections from the indicated groups 8 weeks after tenotomy. The BM areas are expressed as a percentage of the total tendon area. Scale bar: 5 μm. *n* = 5 biological replicates. (**E**–**H**) Representative IF images quantification (right) for SOX9 (red), COL2A1 (green), RUNX2 (red), and OCN (green) of injured tendon sections in the indicated groups after tenotomy, with DAPI counterstaining (blue). Positive staining areas of these proteins were quantified. Scale bar: 5 μm. *n* = 5 biological replicates. (**I** and **J**) TB staining was performed on TDSCs challenged with rmNGF (1, 10, 100 ng/mL), GW (1 μM), or rmNGF (100 ng/mL) + GW (1 μM) in chondrogenic culture for 14 days, compared with control (Con) group and chondrogenesis-alone (Ch) group. (**K** and **L**) Under the aforementioned conditions (**I** and **J**), Western blotting and densitometric quantification (right) of COL2A1 and SOX9 were performed, with β-actin as the loading control. *n* = 3 biological replicates. Data are representative of 2 independent experiments (**B**–**L**). Data were shown as mean ± SD and compared with 1-way ANOVA with Tukey’s multiple-comparison test.

To further delineate the potential role of NGF in promoting traumatic HO, we administered 2 agents via i.p. injection to C57BL/6J mice with tenotomy: rmNGF and the TrkA-specific receptor inhibitor GW441756 (GW) (Figure 4A). After 8 weeks of injections, μCT scans showed a marked increase in ectopic BV in the rmNGF group compared with the control group and a marked decrease in the GW group (Figure 4B). Histological staining analysis of the ectopic bone marrow cavity area demonstrated results consistent with the μCT results (Figure 4, C and D). IF staining analysis of SOX9, COL2A1, RUNX2, and OCN revealed that NGF/TrkA signaling markedly promoted the pathological process of endochondral osteogenesis in traumatic HO (Figure 4, E–H). Chondrogenic differentiation of mesenchymal stem cells (MSCs) or precursor cells is considered a crucial step in the formation of HO (1, 4). In this regard, we induced chondrogenic differentiation in mouse tendon-derived stem cells (TDSCs) and performed costimulation with rmNGF or GW. Unexpectedly, both cellular staining (Figure 4, I and J) and Western blotting (Figure 4, K and L) results demonstrate that the addition of rmNGF or GW did not influence the chondrogenic differentiation of TDSCs.

To address this discrepancy, we examined the expression of NGF and TrkA in the traumatized tendon tissue. Although IF staining revealed abundant expression of NGF in the damaged tissues (Supplemental Figure 1, A and C), TrkA expression was primarily localized to s.c. mucosa and bone marrow cells (Supplemental Figure 1, B and D). Both IF double-staining results (Supplemental Figure 1, E and F) and Western blotting (Supplemental Figure 1, G and H) demonstrate the absence of TrkA expression in TDSCs during the pathogenesis of HO. Thus, NGF cannot effectively couple with TrkA to drive the pathological differentiation of tissue-specific stem cells. Remarkably, the s.c. mucosa and bone marrow cavity are areas known for a high concentration of mast cells (29). Simultaneously, single-cell RNA-Seq (scRNA-Seq) data from the traumatized tissue confirmed that mast cells were the exclusive immune cells expressing TrkA (Supplemental Figure 1I). Given our findings of the activation potential of NGF/TrkA signaling in mast cells, we hypothesized that the binding of NGF to the mast cell surface receptor TrkA may lead to excessive immune response activation, ultimately resulting in HO.

**Figure 5:**
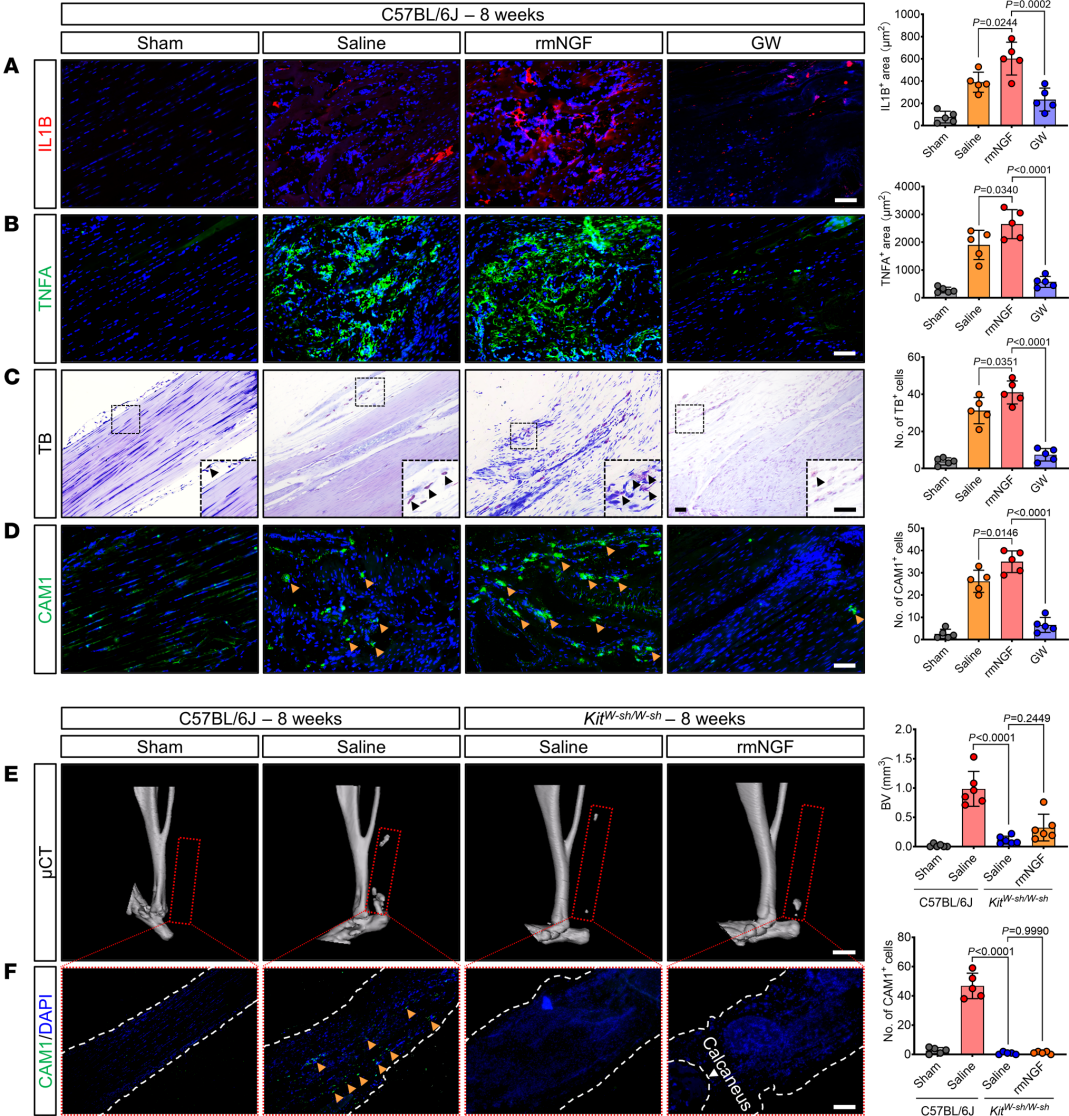
NGF/TrkA signaling exacerbates inflammation associated with mast cell activation in trauma-induced HO. (**A** and **B**) Representative IF staining images and quantification (right) of (**A**) IL1B (red) and (**B**) TNFA (green) in injured tendon sections treated with either rmNGF or GW groups after tenotomy. Black arrows indicate positive cells. Scale bar: 5 μm. *n* = 5 biological replicates. (**C**) Representative TB staining images and quantification (right) of the mast cells in injured tendon sections treated with either rmNGF or GW groups after tenotomy. The total number of mast cells was counted. Black arrows indicate mast cells. Scale bar: 5 μm. *n* = 5 biological replicates. (**D**) Representative IF staining images and quantification (right) of CAM1 (green) in injured tendon sections treated with either rmNGF or GW groups after tenotomy. Yellow arrows indicate positive cells. Scale bar: 5 μm. *n* = 5 biological replicates. (**E**) Representative μCT 3D modeling images of Achilles tendons (sagittal view) in indicated group after tenotomy and quantification (right) of ectopic BV. The red rectangular dashed box represents the reconstruction image of the ectopic bone. Scale bar: 2 mm. *n* = at least 6 biological replicates. (**F**) Representative IF staining images and quantification (right) of CAM1 (green) in Achilles tendon sections of C57BL/6J and *Kit^W-sh/W-sh^* mice treated with or without rmNGF groups after tenotomy, with DAPI counterstaining (blue). The number of CAM1^+^ cells was counted. The white dashed line indicates the margin of the Achilles tendon and calcaneus. Yellow arrows indicate activated mast cells. Scale bar: 20 μm. *n* = 5 biological replicates. All data are representative of 2 independent experiments. Data were shown as mean ± SD and compared with 1-way ANOVA with Tukey’s multiple-comparison test.

Further analysis with IHC staining revealed that i.p. administration of rmNGF in HO-modeled C57BL/6J mice led to a marked increase in IL1B^+^ and TNFA^+^ cells, compared with the saline group, while the GW group showed a marked reduction of them (Figure 5, A and B, and Supplemental Figure 7). These results indicate that NGF/TrkA signaling regulates the local inflammatory response in traumatized tissue. Subsequently, TB staining and IF analysis of CAM1^+^ cells demonstrate that NGF increased the abundance of mast cells in the traumatized tissues and also enhanced their activation (Figure 5, C and D, and Supplemental Figures 7 and 8). Furthermore, i.p. administration of rmNGF was performed in HO-modeled *Kit^W-sh/W-sh^* mice to observe the formation of HO. Although there was an upward trend in HO after mast cell deletion, there was no statistical difference compared with the saline group (Figure 5E). IF staining of CAM1^+^ cells also showed the absence of CAM1 expression in both the saline and rmNGF groups of *Kit^W-sh/W-sh^* mice (Figure 5F). Taken together, these results confirm that NGF/TrkA signaling can upregulate the local inflammatory response through mast cell activation, leading to the pathogenesis of traumatic HO.

### Activated mast cells secrete NT3 to regulate HO progression.

**Figure 6:**
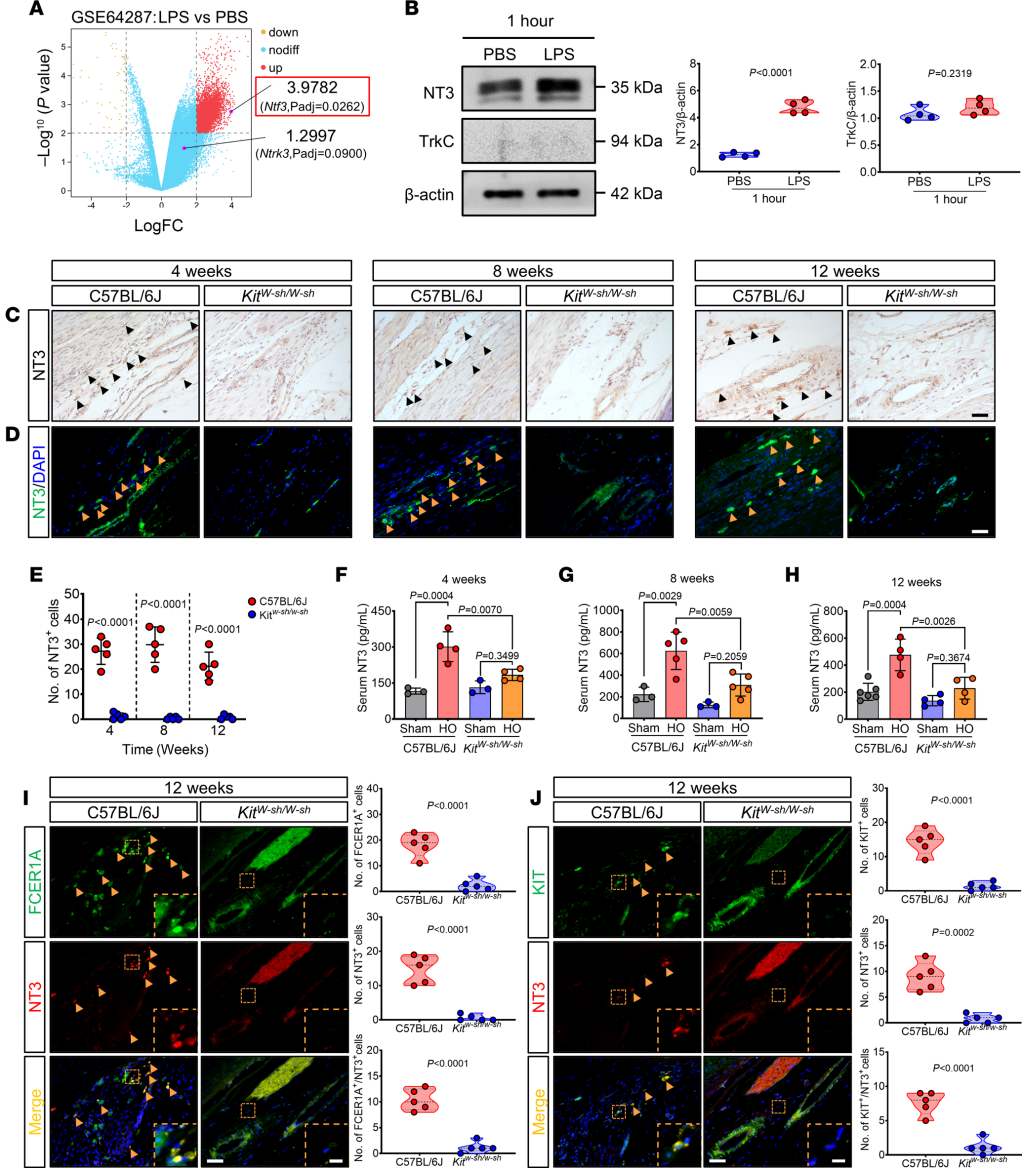
Mast cell–derived NT3 is present throughout the pathogenesis of HO. (**A**) Volcano plot analysis of upregulated (red) and downregulated (yellow) DEGs in BMMCs treated as shown in Figure 3A from the dataset GSE64287. Expression of the *Ntf3* (red box) is significantly increased (*P*_adj_ < 0.05, log_2_FC > 2). (**B**) Western blotting and densitometric quantification (right) of NT3 and TrkC in BMMCs treated with PBS and LPS for 1 hour. β-Actin was used as the loading control. *n* = 4 biological replicates. (**C**–**E**) Representative IHC (**C**), IF staining images (**D**), and quantification (**E**) of NT3 of injured tendon sections in indicated group 4 weeks, 8 weeks, or 12 weeks after tenotomy. Black and yellow arrows indicate NT3^+^ cells. Scale bar: 5 μm. *n* = 5 biological replicates. (**F**–**H**) Serum NT3 levels were measured 4 weeks, 8 weeks, and 12 weeks after HO modeling in C57BL/6J and *Kit^W-sh/W-sh^* mice. *n* = at least 3 biological replicates. (**I** and **J**) Representative IF double-staining images and quantification (right) of FCER1A^+^ (green)/NT3^+^ (red) and KIT^+^ (green)/NT3^+^ (red) cells of injured tendon sections in indicated group 12 weeks after tenotomy, with DAPI counterstaining (blue). The number of positive cells was counted. Yellow arrows indicate mast cells. Scale bar: 5 μm (left) and 1 μm (right). *n* = 5 biological replicates. Data are representative of 2 independent experiments (**B**–**J**). Data were shown as mean ± SD and compared with 2-tailed unpaired Student’s *t* test (**B**, **E**, **I**, and **J**) or 1-way ANOVA with Tukey’s multiple-comparison test (**F**–**H**).

Cytokines released by mast cells are recognized as pivotal regulators of immune responses and potential drivers of abnormal stem cell differentiation (30). Given the role of mast cells in neuroinflammation (29), we posited that they might also release neurotrophins in response to danger signals. Analysis of the microarray dataset (GSE64287) revealed upregulated 3, 519 DEGs (adjusted P [*P*_adj_] < 0.05, log_2_ fold change [log_2_FC] > 2) in LPS-treated mast cells (Figure 6A). Apart from substance P, a neuropeptide known to be secreted by mast cells (Supplemental Figure 12), we observed marked expression of *Ntf3* (the gene encoding NT3) in BMMCs, but *Ntrk3* (the gene encoding TrkC) was not expressed (Figure 6A). These findings were further validated by Western blotting (Figure 6B). To further examine the relation between mast cells and NT3 during traumatic HO, we investigated NT3 expression levels in the serum of C57BL/6J and *Kit^W-sh/W-sh^* mice at different time points after Achilles tenotomy. There was a markedly elevated level of serum NT3 in C57BL/6J mice compared with the sham-operated group but not in *Kit^W-sh/W-sh^* mice. (Figure 6, F–H). IF and IHC staining results support the aforementioned findings (Figure 6, C–E, and Supplemental Figure 9, A and B). IF double-staining experiments revealed the colocalization of FCER1A and KIT with NT3 (Figure 6, I and J). In summary, these data provide evidence that mast cells are capable of secreting NT3, which may have an influence on HO.

Next, we investigated NT3 expression in damaged tendon tissue using IF staining at the early stage of HO onset. The results reveal a remarkable increase in NT3 expression in C57BL/6J mice after 4 weeks of HO modeling compared with the sham-operated group (Supplemental Figure 2, A and C). Furthermore, IHC staining provided insight into the spatial distribution of TrkC expression in traumatized tissue. Damaged tendon and bone cells in the HO-modeled group exhibited marked TrkC expression, which was absent in the sham-operated group (Supplemental Figure 2, B and E). Similarly, the expression of NT3 and TrkC in HO-modeled tissue proteins markedly increased compared with the sham-operated group (Supplemental Figure 2D). During chondrogenic differentiation induction in TDSCs, Western blotting results demonstrate markedly higher expression of TrkC (Supplemental Figure 2F). However, the expression of NT3 was comparatively lower and not statistically significant (Supplemental Figure 2F). Above findings suggest that TDSCs may undergo direct regulation through paracrine NT3 during pathological differentiation. To test this hypothesis, we constructed lentivirus-carrying (LV-carrying) *Ntrk3* knockdown sequences to transfect TDSCs or cocultured with recombinant human NT3 (rhNT3) in a chondrogenic medium. Western blotting and TB staining revealed a reduction in the degree of chondrogenic differentiation of TDSCs in the *Ntrk3* knockdown group, whereas the rhNT3-treated group exhibited an increase (Supplemental Figure 2, G–J). In summary, these findings suggest that mast cell–derived NT3 promotes HO by directly regulating the abnormal chondrogenic differentiation of TDSCs.

### Lipid A in LPS cooperates with NGF in binding to TrkA and enhances its phosphorylation.

**Figure 7:**
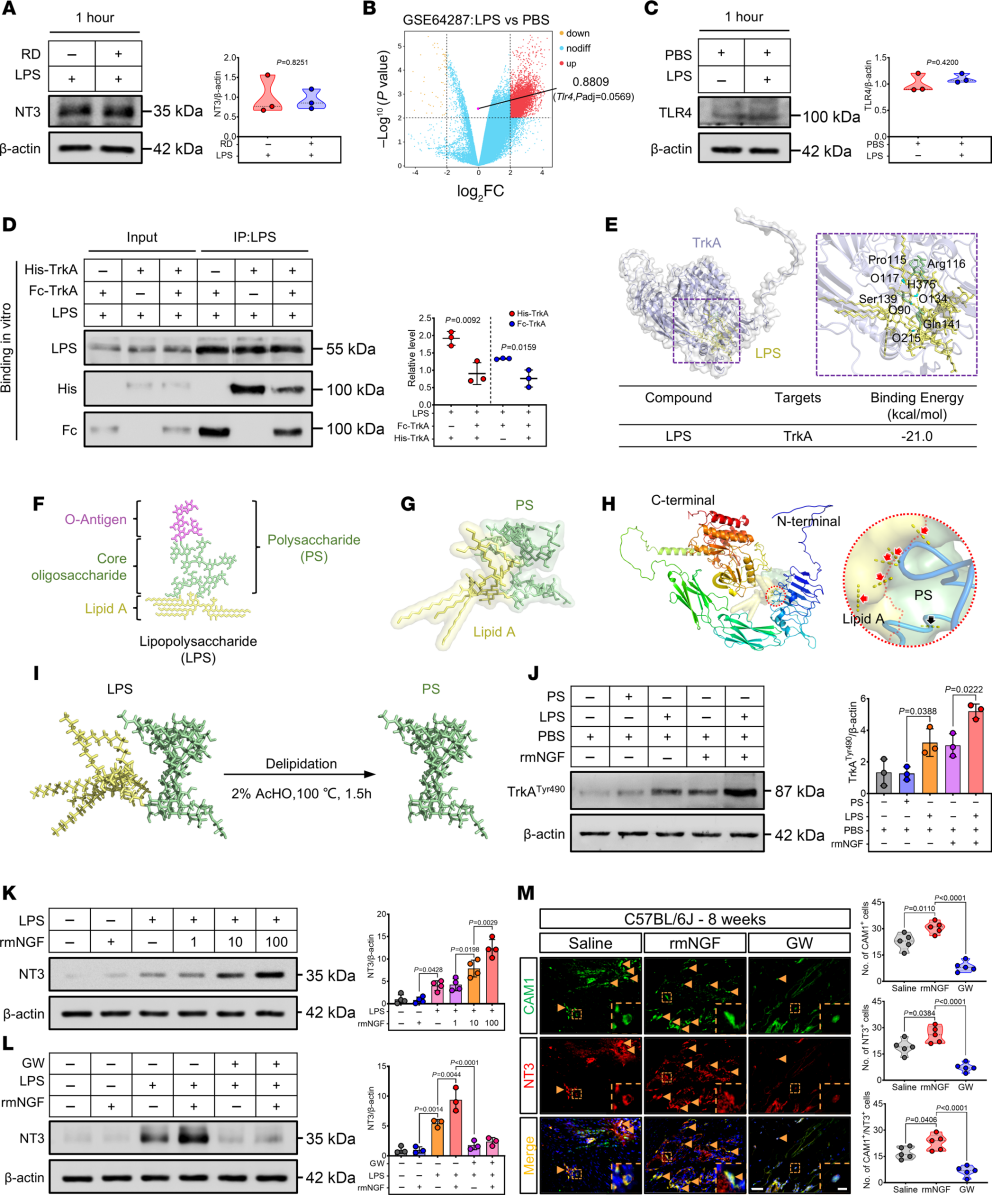
NGF and lipid A in LPS cobind to TrkA, triggering mast cells to secrete NT3 after trauma. (**A**) Western blotting and densitometric quantification (right) of NT3 in BMMCs after LPS (100 ng/mL) or RD (1 μM) treatment for 1 hour, with β-actin as the loading control, *n* = 3 biological replicates. (**B** and **C**) Representative (**B**) expression of Tlr4 (*P*_adj_ > 0.05, log_2_FC < 1) in the GSE64287 dataset, confirmed by (**C**) Western blotting. (**D**) Western blotting and densitometric quantification (right) of LPS competitive binding to His-tagged or Fc-tagged TrkA in vitro (*n* = 3 biological replicates). (**E**) Binding model for LPS (yellow) with TrkA (purple), showing key atoms in LPS (blue sticks) and residues in TrkA (green sticks). (**F**–**I**) Representative (**F**) planar chemical structure, (**G**) lowest free energy 3D structure of LPS, (**H**) hydrogen bonds between LPS and TrkA in lipid A (red arrows) or PS (black arrow), and (**I**) schematic of LPS delipidation strategy. (**J**) Western blotting and densitometric quantification (right) of TrkATyr490 in BMMCs after treatment with LPS, PS, or rmNGF for 1 hour, with β-actin as the loading control, *n* = 3 biological replicates. (**K** and **L**) Western blotting and quantification (right) of NT3 in BMMCs after pretreatment with LPS (100 ng/mL) or PBS, followed by treatment with rmNGF (0–100 ng/mL) or GW (1 μM) for 1 hour, with β-actin as the loading control, *n* = 4 biological replicates. (**M**) IF double-staining images and quantification (right) of CAM1^+^ (green)/NT3^+^ (red) cells in injured tendon sections, with DAPI counterstaining (blue). Scale bar: 5 μm (left) and 1 μm (right), *n* = 5 biological replicates. Data are representative of 2 independent experiments (**A**–**D** and **J**–**M**). Data shown as mean ± SD, compared by Student’s *t* test (**B**–**D**) or 1-way ANOVA (**J**–**M**).

The results of the aforementioned experiments show that LPS-treated mast cells secrete NT3 (Figure 6, A and B). TLR4 is a specific receptor for LPS (31), and its activation may influence NT3 expression in mast cells. To clarify whether mast cell-derived NT3 is regulated by activated TLR4, we inhibited TLR4 activity using the TLR4-specific inhibitor Resatorvid (RD). Afterward, we found NT3 expression was not markedly altered in BMMCs (Figure 7A). Further experiments revealed that neither the gene chip data nor the Western blotting results show marked expression of TLR4 (Figure 7, B and C). These results indicate that LPS-induced secretion of NT3 from mast cells cannot be mediated through activation of TLR4 and that LPS must be acting on a different receptor. Notably, the expression of TrkA was markedly upregulated when mast cells were treated with LPS (Figure 3, B and C), suggesting a potential interaction between LPS and TrkA. Therefore, we conducted an in vitro protein competitive binding assay to confirm this potential interaction. The results demonstrate the existence of mutual binding between LPS and TrkA (Figure 7D).

LPS is a macromolecular antigen primarily composed of lipid and polysaccharide (PS) portions, with the lipid portion consisting of lipid A and the PS portion comprising the O antigen and core PS (32) (Figure 7, F and G). Ligand-protein molecular docking analysis showed that there was a marked mutual binding interaction between LPS and TrkA (Figure 7E). Further docking region analysis revealed that H375, H376, O117, O90, O134 in the lipid A portion of LPS, O125 in the core PS portion, and the N-terminal Pro115, Arg116, Ser139, and Gln141 residues in TrkA were hydrogen bonded to each other (Figure 7, E and H). The above findings indicated that lipid A in LPS was the main component used to bind TrkA, so we chemically removed lipid A from LPS to obtain purified PS molecules (Figure 7I). After coculturing PS, LPS, or rmNGF with mast cells, it was found that delipidated LPS could not induce phosphorylation of TrkA, whereas rmNGF and LPS costimulation markedly activated TrkA (Figure 7J). This suggests that Lipid A in LPS acts as a potential costimulatory factor by cooperating with NGF to bind to TrkA, thereby triggering an increased level of TrkA phosphorylation. This may be the reason why NGF combined with LPS can markedly promote the formation of traumatic HO in vivo (Supplemental Figure 3).

### NGF-activated mast cells release NT3 to promote trauma-induced HO in vitro and in vivo.

**Figure 8:**
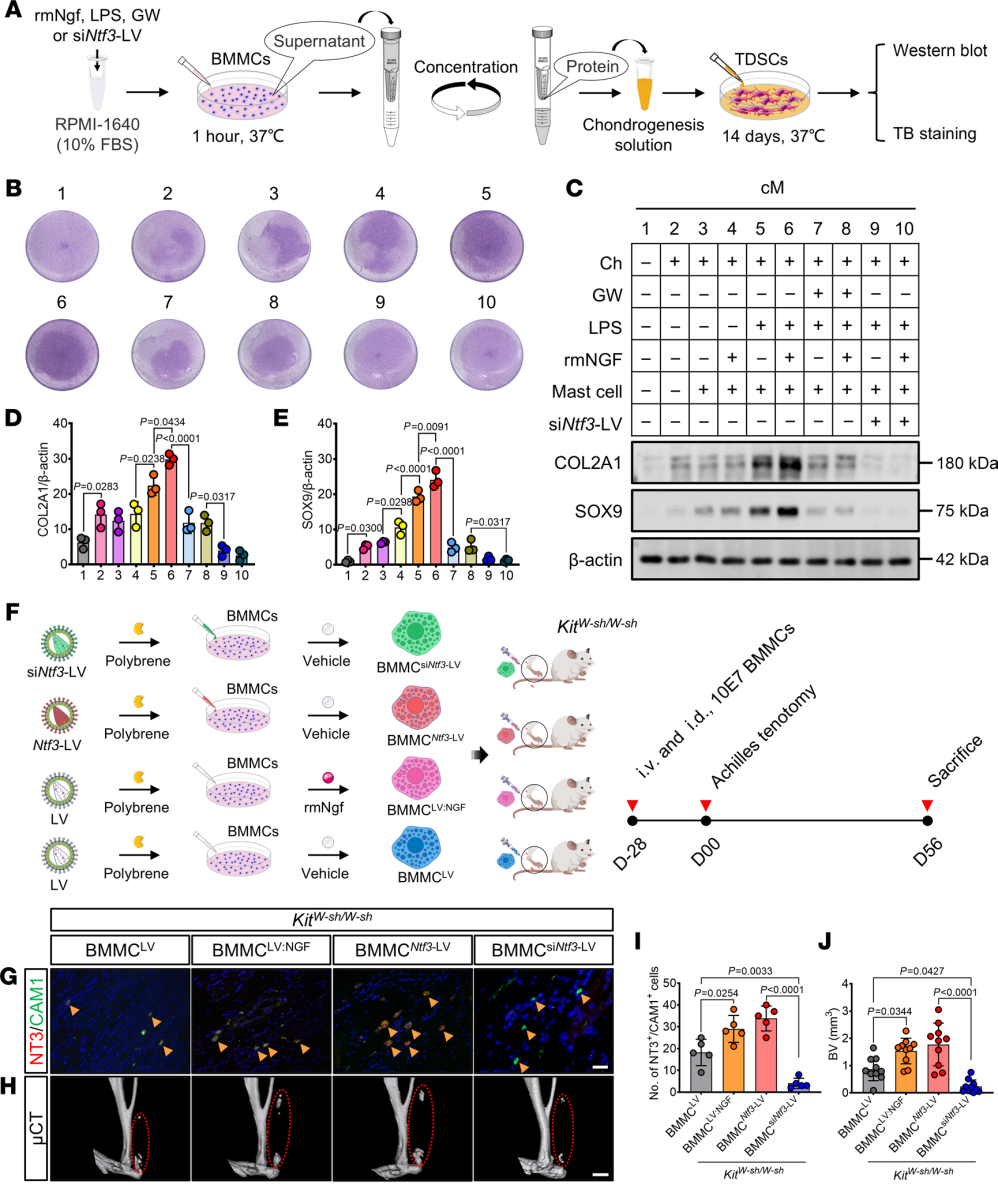
NGF-activated mast cells release NT3 to promote traumatic HO. (**A**) Schematic representation of the acquisition of concentrated conditioned medium (cM). (**B**–**E**) Representative TB staining (**B**), Western blotting (**C**), and densitometric quantification of COL2A1 and SOX9 (**D** and **E**) were performed on TDSCs after 14 days of induction culture, following the experimental procedure described in **A**. In Western blotting, β-actin was used as the loading control. *n* = at least 3 biological replicates. (**F**) Schematic representation of experimental protocol of i.v. and i.d. transfer of BMMC^LV^, BMMC^LV: NGF^, BMMC^Ntf3-LV^, and BMMC^si*Ntf3*-LV^ into *Kit^W-sh/W-sh^* mice for the generation of HO mouse model. (**G** and **I**) Representative IF double-staining images and quantification (**I**) of NT3^+^ (red)/CAM1^+^ (green) cells of injured tendon sections in indicated group 8 weeks after tenotomy, with DAPI counterstaining (blue). The number of positive cells was counted. Yellow arrows indicate mast cells. Scale bar: 5 μm. *n* = 5 biological replicates. (**H** and **J**) Representative μCT 3D modeling images of Achilles tendons (sagittal view) in indicated group after tenotomy and quantification (**J**) of ectopic BV. Red dashed ovals represent the reconstruction image of the ectopic bone. Scale bar: 2 mm. *n* = at least 10 biological replicates. Data are representative of 2 independent experiments (**B**–**E** and **G**–**J**). Data were shown as mean ± SD and compared with 1-way ANOVA with Tukey’s multiple-comparison test.

Further cell induction experiments showed that NGF promoted NT3 secretion from mast cells in a dose-dependent manner in the presence of LPS (Figure 7, K and L), confirming the involvement of NGF/TrkA signaling in NT3 secretion by mast cells in vitro. In animal experiments, we investigated the coexpression of CAM1 and NT3 in tendon tissues of HO-modeled C57BL/6J mice following i.p. injections of saline, rmNGF, and GW. We observed a marked increase in CAM1^+^/NT3^+^ cell numbers in the rmNGF-injected group and a marked decrease in the GW-injected group, both compared with the saline group (Figure 7M). As a whole, these findings indicate that the activation of mast cells through NGF/TrkA signaling contributes to the high expression of NT3. To clarify the role of NT3 secreted by NGF-activated mast cells in aberrant cell fate, we collected concentrated conditioned medium (cM) from different treatments and cultured TDSCs using it for 14 days (Figure 8A). Cellular TB staining demonstrated that the protein factors secreted by mast cells treated with both rmNGF and LPS markedly enhanced the chondrogenic differentiation of TDSCs (Figure 8B). Western blotting confirmed TB staining results (Figure 8, C–E). Specific knockdown of *Ntf3* expression in mast cells markedly inhibited the chondrogenic differentiation of TDSCs (Figure 8, B–E).

The NT3 neutralizing antibody experiment further confirmed the role of NT3 in promoting the chondrogenic differentiation of TDSCs (Supplemental Figure 5 and Supplemental Tables 3 and 4). In animal experiments, we successfully conducted mast cell reconstitution in *Kit^W-sh/W-sh^* mice through tail i.v. and i.d. injections of BMMCs (Figure 8F and Supplemental Figure 4, A–C). Following mast cell reconstitution in *Kit^W-sh/W-sh^* mice, ectopic BV exhibited a notable increase compared with the control group (Supplemental Figure 4D). Moreover, injection of *Kit^W-sh/W-sh^* mice with BMMCs overexpressing *Ntf3* or a mixture of BMMCs and rmNGF resulted in markedly higher BV, compared with the group injected with BMMCs alone (Figure 8, G–J). However, injection of *Kit^W-sh/W-sh^* mice with BMMCs knockdown *Ntf3* showed a marked decrease in ectopic BV (Figure 8, G–J). In conclusion, these experimental findings confirm that NT3 secreted by NGF-regulated mast cell induced the formation of traumatic HO.

### Analysis of single-cell data and human tissue samples revealed the presence of NGF-activated mast cells secreting NT3 in the pathological process of HO.

**Figure 9:**
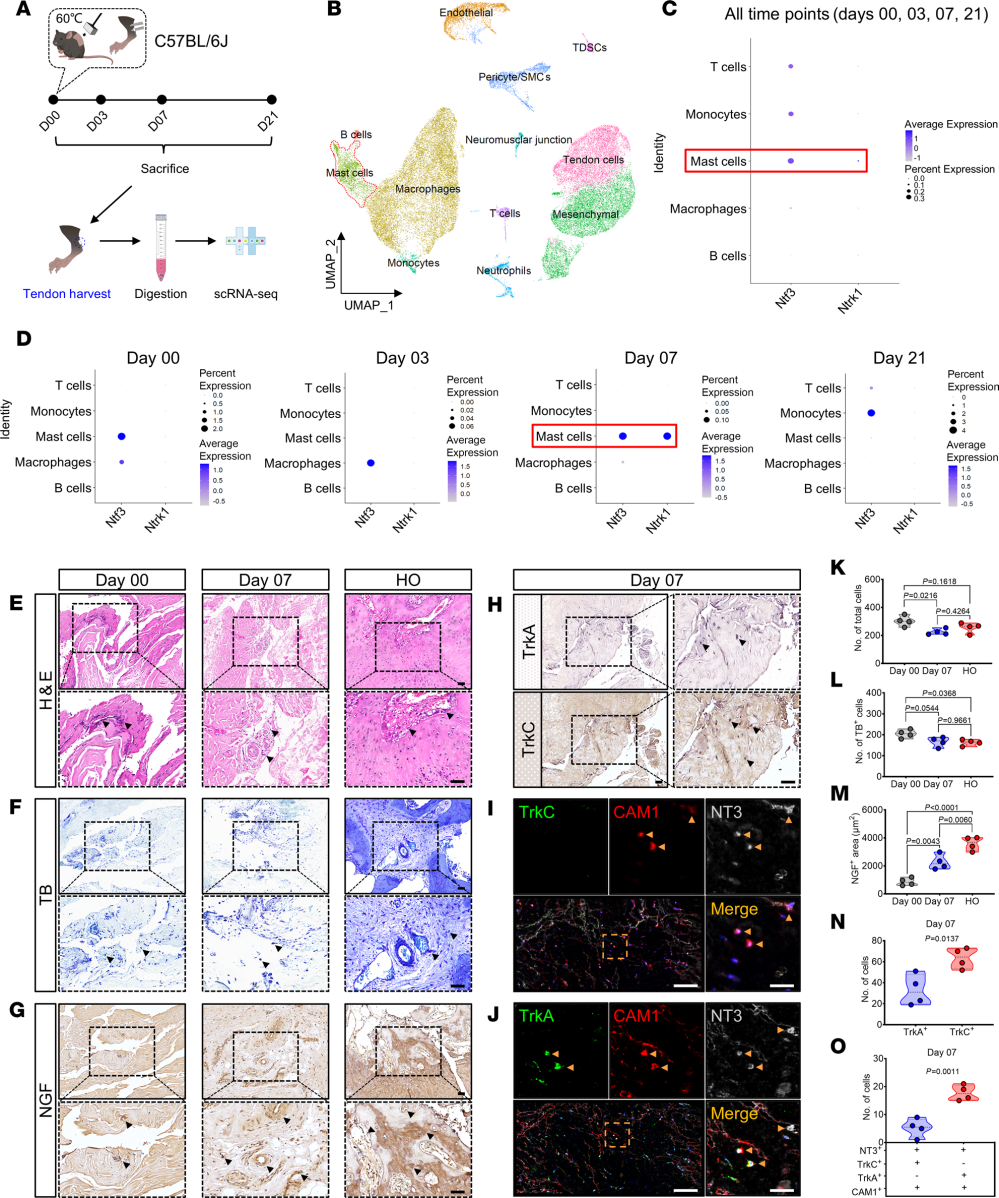
scRNA-Seq analysis and human traumatic tissue assay confirm the involvement of NGF/TrkA signaling and mast cell–derived NT3 in HO. (**A**) Schematic diagram of the experimental workflow for the scRNA-Seq dataset GSE126060. (**B**) UMAP plots revealed 12 distinct cell clusters, including mast cells (red dashed line). (**C**) Bubble plot showing the expression of *Ntf3* and *Ntrk1* in 5 types of immune cells. (**D**) Seven days after HO induction, mast cells exhibited a high expression level of *Ntf3* and *Ntrk1* (red box). (**E**, **F**, **K**, and **L**) Representative H&E (**E**), TB staining images (**F**), and quantification of the total cells (**K**) and mast cells (**L**) count in human tendons at 0 and 7 days after trauma, as well as in HO tissues. Black arrows indicate inflammatory cell and mast cell. Scale bar: 5 μm. *n* = 4 biological replicates. (**G**, **H**, **M**, and **N**) Representative IHC staining images (**G** and **H**) and quantification of NGF, TrkA, and TrkC expression (**M** and **N**) in human ligaments at 0 and 7 days after trauma, as well as in HO tissues. Black arrows indicate positive areas. Scale bar: 5 μm. *n* = 4 biological replicates. (**I**, **J**, and **O**) Representative polychromatic immunofluorescence staining images (**I** and **J**) and quantification of TrkC^+^/TrkA^+^ (green), CAM1^+^ (red), and NT3^+^ (silver) cells (**O**) in human ligaments at 7 days after trauma, with DAPI counterstaining (blue). The number of colocalized positive cells was counted. Yellow arrows indicate mast cells. Scale bar: 80 μm (left) and 20 μm (right). *n* = 4 biological replicates. Data are representative of 2 independent experiments (**E**–**O**). Data were shown as mean ± SD and compared with 1-way ANOVA with Tukey’s multiple-comparison test (**K**–**M**) or 2-tailed unpaired Student’s *t* test (**N** and **O**).

Due to our previous study highlighting the importance of NT3 as a crucial link between neurology and immunity in the pathogenesis of traumatic HO, we analyzed the single-cell dataset to identify immune cells capable of expressing *Ntf3* (Figure 9, A and B). We found that *Ntf3* was expressed by mast cells, monocytes, T lymphocytes, and macrophages, but only mast cells expressed *Ntrk1* (Figure 9C). This reinforces the critical role of NGF/TrkA signaling in mast cells for the regulation of NT3 secretion. Subsequent analysis of *Ntf3* and *Ntrk1* expression in mast cells at different time points (days 0, 3, 7, and 21) following HO modeling revealed the coexpression of both genes on the seventh day after injury. (Figure 9D). This indicates that NT3 secreted by NGF-activated mast cells at this specific time point directly participates in the regulation of abnormal differentiation of TDSCs.

Finally, we performed histological analyses on injured human tissue samples. Staining with H&E (Figure 9, E and K) and TB (Figure 9, F and L) revealed marked infiltration of inflammatory cells and mast cells in the ligament on days 0 and 7 after trauma as well as in the ligament tissue where HO occurred. IHC staining showed that NGF expression increased with time after trauma (Figure 9, G and M). Importantly, marked differences were observed in the expression of TrkA and TrkC within the same tissue region, with TrkA expression being scarce 7 days after injury, while TrkC expression showed a marked increase (Figure 9, H and N). This suggests that NT3/TrkC signaling plays a crucial regulatory role in the aberrant differentiation of tissue-specific stem cells. Moreover, polychromatic IF colocalization analysis of CAM1, NT3, and TrkA demonstrated that activated mast cells express TrkA and secrete NT3 during the pathogenesis of HO in human tissue (Figure 9, I, J, and O). These findings provide further support for our observations.

## Discussion

In the present study, we used transgenic and surgical animal models to demonstrate the pivotal role of mast cells during the HO pathogenesis, emphasizing their contribution to HO within traumatized soft tissue. Mast cells exhibit neurotropism in HO-diseased tissues, which correlates with the secretion of neurotrophic factors such as NGF (8, 9, 12, 29). Previous studies showed that NGF can promote traumatic HO formation, but direct evidence of its ability to regulate the aberrant differentiation fate of tissue-specific stem cells is still lacking (14). Our findings suggest that NGF does not directly modulate this process. Instead, it activates mast cells and triggers the secretion of cytokines, which in turn promote chondrogenic differentiation of TDSCs during HO formation. This provides initial insights into the role of neuroinflammation in governing the pathogenesis of traumatic HO.

Several studies have indicated that soft tissues without peripheral innervation are unlikely to develop HO, even when subjected to trauma (8, 14, 33, 34). Through TrkA inhibition, our results reaffirm the crucial role of neural ingrowth driven by NGF for HO. NGF is not only instrumental in facilitating sensory nerve invasion, but it also modulates the activation, proliferation, and chemotactic aggregation of mast cells (15, 19). Our results suggest that NGF can promote traumatic HO formation by stimulating mast cell activation in the presence of LPS. Current literature indicates that numerous antigenic substances, including LPS and lysophosphatidylserine, can synchronize mast cell activation (18, 35). LPS acts as the primary immunogen, triggering an inflammatory response in soft tissue trauma (36). It induces mast cells to release inflammatory factors but does not result in their degranulation (28, 37, 38). However, the mechanism by which LPS synergizes with NGF to induce cytokine secretion from mast cells is unclear. Our study reveals that lipid A in LPS markedly enhanced NGF-induced phosphorylation of TrkA upon binding to TrkA. This finding aligns with studies emphasizing the pathogenicity of LPS primarily in its lipid fraction (31). Bacterial infections frequently occur after soft tissue trauma, and the use of vancomycin for antiinflammatory debridement has been shown to mitigate HO (23, 39). This effect may be attributed to the attenuation of the NGF-induced cytokine storm, which results from the reduced levels of LPS following bacterial suppression. A recent study has provided evidence that bacterial LPS can exacerbate the development of neurogenic HO (25), providing support for our hypotheses.

An important question is which cytokines, secreted by mast cells, modulate the chondrogenic differentiation of stem cells in situ. While the neuroendocrine function of mast cells is acknowledged (40), the specific cytokines influencing stem cell differentiation are yet to be identified. Therefore, we probed the gene expression profiles in LPS-treated mast cell microarray data. Beyond established neurogenic cytokines like substance P and calcitonin gene-related peptide that amplify HO pathogenesis (41, 42), we noted an overexpression of *Ntf3* in mast cells. In addition, our previous studies demonstrated the important role of NT3 in promoting traumatic HO pathogenesis (21, 43). Moreover, scRNA-Seq identified mast cells as the dominant NT3-producing immune cells in HO. In the early stages of traumatic HO, TrkC was widely expressed in tendon cells, TDSCs, and chondrocytes, consistent with our previous observations (21). Silencing *Ntrk3* markedly curtailed the chondrogenic differentiation of TDSCs, suggesting a direct involvement of NT3/TrkC signaling in aberrant cell fate differentiation during cartilaginous phases of traumatic HO. Furthermore, the angiogenic potential of NT3 deserves attention (44, 45). The collaborative action of nerves and vessels is paramount for HO progression (46). Mast cell–derived proteases, such as chymase or tryptase, are involved in matrix degradation and stimulate vascular invasion (29). Thus, mast cell–released NT3 might initiate vascular invasion, culminating in HO growth.

The present study has several additional limitations. First, while mast cell deletion led to a marked reduction in HO formation, it did not entirely inhibit the process, indicating that HO development is not driven by a single inflammatory cell type. Our previous study demonstrated a marked reduction in traumatic HO following macrophage clearance (43). Additionally, Convente et al. reported that FOP was reduced by approximately 50% with the depletion of either mast cells or macrophages alone and by up to 75% when both were depleted simultaneously (10). This suggests that the interplay among multiple inflammatory cells may play a more substantial role in traumatic HO progression. Second, scRNA-Seq data reveal temporal differences in TrkA expression within mast cells during various stages of the inflammatory response, with the most pronounced expression observed on day 7 after injury. This time point may represent a pivotal shift from an acute inflammatory phase to a reparative phase where neurotrophic signaling becomes more influential (1). Although we have confirmed TrkA expression in mast cells within human traumatic tendon tissue on this day, we did not assess expression at other time points. The temporal variation in neurotrophic signal sensing by mast cells could have marked implications for the aberrant stem cell differentiation (15). Further exploration is needed to elucidate the details of their communication.

In the context of inhibiting HO formation, while NGF/TrkA signaling inhibition or mast cell activation suppression shows promise (11, 14), current suppressant drugs have notable limitations. Tanezumab, an emergent NGF-neutralizing antibody, has shown effectiveness against arthritis pain and other symptoms, but its clinical viability is questionable due to marked adverse reactions at high doses and limited effectiveness at lower doses (47, 48). Although the mast cell inhibitors imatinib and cromolyn have proven effective in decreasing HO (11, 49), their clinical use is constrained by their lack of specificity, which might result in unintended effects on other physiological functions (50, 51). Our research indicates that mast cell–derived NT3 is crucial to early HO formation. Mast cells are known to accumulate in inflamed tissues (12); thus, selectively targeting mast cell NT3 secretion after trauma could provide localized HO inhibition at distinct tissue sites. This approach might reduce the broader adverse effects seen with extensive mast cell suppression. Consequently, curtailing NT3 secretion by mast cells is a promising therapeutic approach for specifically halting HO in traumatized soft tissues.

## Methods

Supplemental Methods are available online with this article.

### Sex as a biological variable.

Our study examined male and female animals, and similar findings are reported for both sexes.

### Human trauma and HO specimens.

Eight traumatic human Achilles tendon specimens were collected from patients with ruptures caused by sports injuries and accidents. Additionally, 4 nongenetic HO specimens were obtained from patients with ossification of the posterior longitudinal ligament (OPLL). The above specimens were all retrieved from the surgical pathology archives of Nanfang Hospital. A summary of patient demographics is provided in Supplemental Table 6.

### Animals.

Mast cell–deficient (*Kit^W-sh/W-sh^*, C57BL/6J background) mice (52) were acquired from The Jackson Laboratory (stock no. 012861), and WT (C57BL/6J) mice were obtained from the Medical Animal Center of Southern Medical University (Supplemental Table 5). C57BL/6J mice as controls were not littermates. The 2 strains of mice were bred and maintained independently. All mice were housed in an SPF-grade facility with a temperature of 18°C–22°C, relative humidity of 50% (±20%), and a 12-hour light-dark cycle. Unless specified otherwise, the mice had ad libitum access to food and water. Mice of either sex aged 8-10 weeks in the same experimental group were randomly allocated to different treatments or experimental procedures.

### Establishment of animal models.

A trauma-induced HO model was created by completely severing the Achilles tendon. Our prior reports describe the method in detail (20, 53). In brief, the experimental group underwent midpoint Achilles tenotomy on the right hind limbs through a posterior approach. The incision was routinely closed with an interrupted 5-0 silk suture, whereas the control group underwent only a skin incision. At 4, 8, and 12 weeks after operation, all animals were sacrificed by the administration of a fatally high dose of anesthetics.

### In vivo treatment with rmNGF and GW.

C57BL/6J mice received an i.p. injection of rmNGF and GW (Supplemental Table 7) 3 days before tenotomy, followed by daily administration for 8 weeks. The rmNGF (450-34, Peprotech) was dissolved in distilled water, combined with alginate (HY-CP001, MCE) for a stock solution, and diluted in saline for i.p. administration at 4 ng/g/day (54, 55). GW was prepared in DMSO, diluted with saline, and administered i.p. at 10 μg/g/day (56, 57). The vehicle control treatment involved a 200 μL injection of 2% DMSO/saline.

### IHC and histological analysis.

Specimens were harvested and placed in 4% paraformaldehyde at 4°C for 24–48 hours. The samples were decalcified using 0.5M EDTA for 1 month at room temperature (RT). After dehydration and transparency processes, specimens were embedded and sectioned at a thickness of 4 μm. Next, sections were dewaxed and hydrated for staining. Histological stains including H&E (Ab245880, Abcam), SOFG (G1371, Solarbio), Masson’s (G1346, Solarbio), ALP (C3206, Beyotime), and TB (G3660, Solarbio). All staining procedures were performed in accordance with the instructions provided in the staining kits. For IHC, sections underwent antigen retrieval using proteinase K at 37°C for 10–15 minutes. Endogenous peroxidase was inactivated with 3% hydrogen peroxide at RT for 15 minutes. The sections were then blocked using normal goat serum (AR0009, Boster) for 1 hour, followed by overnight incubation at 4°C with primary antibodies (Supplemental Table 7) in a humidified chamber. The following day, slides were exposed to the appropriate horseradish peroxidase–conjugated (HRP-conjugated) secondary antibodies for 1 hour at RT. The sections were then stained using the 3,3’-diaminobenzidine chromogenic staining kit (ZLI-9018, ZSGB-BIO). After completion of staining, images were captured using an orthogonal white light microscope (Axioscope 5, Jena) and analyzed using ImageJ software (NIH; version 1.53a).

### IF and polychromatic IF.

Mouse tendon sections were deparaffinized, subjected to antigen retrieval, blocked with goat serum for 1 hour, and then incubated overnight at 4°C with primary antibodies (Supplemental Table 7) in a humidified chamber. The following day, slides were incubated with appropriate fluorescent secondary antibodies (Supplemental Table 7) for 1 hour at RT, and subsequently mounted with DAPI mounting solution (P0131, Beyotime). Images of these sections were captured via upright fluorescence microscopy (Axioscope 5, Jena). Human tissue sections were processed according to the instructions of the 4-color multiplex fluorescence IHC staining kit (abs50012, Absin). Sections were blocked with goat serum before antibody incubation. Antibodies used included CAM1, TrkA, and NT3 (Supplemental Table 7). Nuclei were stained with DAPI before sealing. All sections were scanned using a digital pathology scanner (KF-FL-040, Ningbo).

### Microarray data analysis.

Microarray data for this study were retrieved from the Gene Expression Omnibus (GEO) database, including 6 BMMC samples from C57BL/6J mice, with 3 PBS-treated and 3 LPS-treated (accession no. GSE64287) (26). Data were analyzed using GeneSpring GX (Agilent), as described in the online repository. All fold change data were log_2_ transformed, and *P* values were adjusted to control the FDR through the Benjamini-Hochberg procedure. Further bioinformatics analyses were performed with R and Bioconductor packages. Volcano plots depicting the DEGs were generated by the “ggplot2” R package (58).

### scRNA-Seq data analysis.

The scRNA-Seq data were sourced from the GEO database (accession no. GSE126060) and included samples from C57BL/6J mice subjected to a HO model (30% dorsal burn and Achilles tenotomy). Samples from the injury site were harvested via microdissection on days 3, 7, and 21 after injury and compared with baseline uninjured tissue from the same site (day 0) (14). Data were processed with the R-based Seurat package (59). Cells with ≤ 500 genes per cell and with a mitochondrial read content of ≥ 15% were excluded. Subsequent analyses included normalization, identification of highly variable genes, scaling by the number of unique molecular identifiers (UMIs) to account for sequencing depth and batch effects, dimensionality reduction using principal component analysis (PCA) and uniform manifold approximation and projection (UMAP) (60, 61), unsupervised clustering, and identification of differentially expressed, cell type-specific markers. Sample comparison was performed using Seurat’s embedded canonical correlation analysis function. Following identification of common sources of variation, cells from the 2 datasets were aligned and clustered using unsupervised clustering. The negative binomial test was used to identify DEGs between aligned clusters.

### Isolation and identification of BMMCs and TDSCs.

BMMCs were generated as previously described (26). Briefly, bone marrow cells were harvested and resuspended in complete RPMI1640 medium (11875093, Thermo Fisher Scientific) supplemented with 10 ng/mL recombinant murine IL-3 (rmIL-3) and 10 ng/mL recombinant murine stem cell factor (rmSCF) for induction culture. The medium was refreshed every 3 days for at least 4 weeks. TDSCs, which are multipotent stem cells from tendon tissues, were extracted as per previous reports (62, 63). These cells have shown the capacity to differentiate into osteogenic and chondrogenic lineages upon neuroendocrine factors and may contribute to HO pathogenesis (43, 64, 65). After culture, flow cytometry identified BMMCs and TDSCs, while cellular TB and IF staining further characterized them (Supplemental Figure 10).

### Flow cytometry.

BMMCs and TDSCs were isolated and transferred into tubes, ensuring a cell density of at least 1 × 10^6^ cells/mL. The cells were blocked with anti–mouse CD16/32 and subsequently stained with FCER1A, KIT, SSEA4, OCT4, CD34, and CD106 antibodies (Supplemental Table 7). Following staining and washing, the samples were processed on an LSRFortessa (BD Biosciences) for analysis. Data analysis was performed with FlowJo software (version 10.6.2).

### Molecular docking analysis of LPS and TrkA.

The 3D structure of the small-molecule ligand LPS (CID 53481793) was downloaded from PubChem (https://pubchem.ncbi.nlm.nih.gov/), and the protein TrkA (AF-Q3UFB7-F1-model_v4) was downloaded from the AlphaFold Protein Structure Database (https://alphafold.ebi.ac.uk/) The binding pattern between LPS and TrkA was predicted using the ligand-protein docking method in Autodock Vina (version 1.1.2.) (66). Finally, PyMOL (version 1.7.2.1) was used for interaction analysis of the protein-ligand complex.

### LPS delipidation and isolation of the PS.

A LPS sample was delipidated with a 2% AcOH aqueous solution at 100°C for 1.5 hours (67). The lipid precipitate was removed by centrifugation (12,000*g*, 30 minutes), and the carbohydrate portion was fractionated by Gel Permeation Chromatography on a column of Sephadex G-50 Superffne (G5050, MilliporeSigma) in 0.05M pyridinium acetate buffer (pH 4.5), monitored with a differential refractometer (Knauer) to obtain PS.

### Lentiviral-based transfection and delivery.

Lentiviral vectors encoding *Ntf3*, small interfering RNA targeted to *Ntf3*/*Ntrk3* (si*Ntf3*/si*Ntrk3*-LV), and nonspecific control LV vectors were synthesized by Tsingke. The relevant sequences are shown in Supplemental Tables 1 and 2. LV transfection was performed to either overexpress or knockdown NT3 in BMMCs and to knockdown TrkA in TDSCs. In brief, both TDSCs and BMMCs were seeded in 6-well plates at a density of 1×10^5^. Cells were then transfected with *Ntf3*-LV, si*Ntf3*-LV, si*Ntrk3*-LV, and LV in the presence of polybrene (Tsingke). The transfection efficiency of the lentiviral vector was confirmed using phase contrast and fluorescence microscopy (Olympus IX53), and the efficiency of LV-mediated overexpression of NT3 or downregulation of NT3/TrkC was confirmed by Western blotting analysis (Supplemental Figure 11).

### BMMC activation and TDSC chondrogenic differentiation.

To clarify the relation between NGF/TrkA signaling and mast cell activation, BMMCs were seeded into 12-well plates in duplicate and cultured at 37°C with 5% CO_2_ for 24 hours. Before experimentation, BMMCs were starved in a medium without rmIL-3/rmSCF for 12 hours and then cultured with PS (100 ng/mL), LPS (100 ng/mL), rmNGF (1, 10, and 100 ng/mL) + LPS (100 ng/mL), LPS (100 ng/mL) + GW (1 μM), LPS (100 ng/mL) + RD (1 μM), or rmNGF (100 ng/mL) + LPS (100 ng/mL) + GW (1 μM) for 1 hour. Subsequently, mast cell activation was assessed via TB staining and ELISA, while the expressions of TLR4, NT3, TrkC, NGF, and TrkA in BMMCs were examined by Western blotting.

TDSCs were cultured in 6-well plates (1×10^5^ cells/well) at 37°C and 5% CO_2_ until reaching 90% confluency to evaluate chondrogenic differentiation. Then, the culture medium was replaced with a chondrogenic medium (low-glucose DMEM supplemented with 50 μg/mL ascorbic acid, 10 nM dexamethasone, 1:100 ITS-premix, and 10 ng/mL TGFB3) (63). For analysis of the chondrogenic effects of NGF/TrkA and NT3/TrkC signaling, TDSCs were cultured in a chondrogenic medium with rmNGF (1, 10, and 100 ng/mL), rhNT3 (100 ng/mL), GW (1 μM), or si*Ntrk3*-LV until the end of the culture period day 14, with medium refreshed every 2–3 days. The control group was chondrogenic induction alone, and a standard medium culture group assessed chondrogenic induction effectiveness.

### In vitro experiments with the concentrated cM.

A concentrated cM was prepared as follows. An equal number of BMMCs were treated with rmNGF (100 ng/mL), LPS (100 ng/mL), or GW (1 μM) or were transfected with si*Ntf3*-LV for 1 hour. After cell debris removal, the supernatant was processed through ultrafiltration tubes (UFC8100, Amicon Ultra-4, MilliporeSigma) and was concentrated and desalted according to the manufacturer’s instructions. To eliminate LPS and LV, the supernatant was successively filtered with Ultra-100 kDa and Ultra-10 kDa tubes to obtain the purified proteins derived from BMMCs. Then these proteins were redissolved in a chondrogenesis solution to obtain cM. A control cM was produced similarly but with no BMMCs involved. When TDSCs in 12-well plates achieved over 90% confluence, the high-glucose complete DMEM was replaced with a different cM. The effects of chondrogenic induction were assessed against a standard medium culture control.

### Mast cells reconstitution in Kit^W-sh/W-sh^ mice.

BMMCs were cultured from C57BL/6J mice and adoptively transferred into *Kit^W-sh/W-sh^* mice. BMMCs were transfected with empty control LV (BMMC^LV^), si*Ntf3*-LV(BMMC^si*Ntf3*-LV^),and *Ntf3*-LV (BMMC^Ntf3-LV^). One group of BMMC^LV^ was supplemented with 100 ng/mL of rmNGF (BMMC^LV: NGF^). A total of 1 × 10^7^ BMMCs in 200 μL PBS were adoptively transferred into *Kit^W-sh/W-sh^* mice via tail i.v. and i.d. injection to systemically reconstitute mast cells. In the meantime, *Kit^W-sh/W-sh^* mice received vehicle (PBS) only as the control. Mast cell–reconstituted mice were used to generate a trauma-induced HO model by using the same protocol as described above. Injured tendons were collected 8 weeks after HO modeling and then stained with TB to confirm the presence of mast cells.

### Cell staining.

TB staining was used to assess TDSC chondrogenesis and mast cell degranulation. In brief, after washing with PBS, the different treatment TDSCs were fixed with 4% paraformaldehyde at 4°C for 1 hour and then incubated with TB solution (G2543, Solarbio) for 30 minutes. Excess dye was removed with PBS, and images were captured using a scanner (HP ScanJet Pro 3600 f1). BMMCs, costimulated by rmNGF and LPS for 1 hour, were placed onto slides, stained with TB for 30 seconds, decolorized with 95% alcohol, and observed under a light microscope.

### Western blotting.

Proteins were extracted from cells and tissues using cold RIPA buffer (P0013B, Beyotime) supplemented with phenylmethanesulfonylfluoride (PMSF). They were separated via 8%–12% gradient sodium dodecyl sulfate-polyacrylamide gel electrophoresis (SDS-PAGE) and transferred to nitrocellulose (NC) membranes. These membranes were blocked with 5% nonfat milk for 1 hour at RT and were then incubated overnight with primary antibodies (Supplemental Table 7). After washing, the membranes were treated with HRP goat anti–mouse/rabbit IgG for 1 hour at RT. Protein bands were visualized using an ECL kit (P0018S, Beyotime), images were captured with a Tanon 5200 Imaging System, and band intensities were quantified with ImageJ software.

### Ligand-protein competitive binding assays.

rmHis-TrkA, rmFc-TrkA, or a combination of rmHis-TrkA and rmFc-TrkA, each at a concentration of 10 μM, were separately incubated with 10 μM LPS in a final volume of 500 μL for 30 minutes at RT. The proteins were then immunoprecipitated with anti-His or anti-Fc antibodies (Supplemental Table 7), followed by incubation with Protein A/G-agarose (20422, Thermo Fisher Scientific) overnight at 4°C with rotation. After 5 washes with lysis buffer, the immune complexes were dissolved in 2× SDS-PAGE loading buffer (P0015B, Beyotime), and the precipitated LPS and His-TrkA or LPS and Fc-TrkA were detected by Western blotting.

### ELISA.

The concentration of NT3 in mouse serum and the levels of HA and Hex in mast cell supernatants from various treatment groups were determined using designated kits (LV30412, LV30665, LV30666; Animal Union) following the manufacturer’s instructions. Absorbance was measured at 450 nm with an iMark Microplate Absorbance Reader (Bio-Rad). The Hex release rate was quantified via ELISA in both the supernatant and cell lysate. Cells were lysed with 1% Triton X-100 (MilliporeSigma), and the resulting lysate was centrifuged at 300*g* for 5 minutes. The release rate was then calculated using the formula: Hex release rate = A / (A + B) × 100%, where A is the level of Hex in the cell lysate supernatant and B is the level of Hex in the cell lysate.

### μCT imaging and analysis.

Mouse hindlimbs were harvested and imaged using the Latheta LCT 200 system (Hitachi Aloka Medical Ltd.), operating with a pixel size and slice thickness of 48 μm. After scanning, parameters such as heterotopic BV, BA, BMC, and BMD were analyzed, and 3D reconstruction was performed using LaTheta software (version 1.3).

### Statistics.

Evaluations were undertaken by researchers blinded to the study design, with each experiment incorporating a minimum of 3 independent samples. Numerical values signified significant intergroup differences. Data were represented as mean ± SD and analyzed with GraphPad Prism 9.0 software. Normality and variance homogeneity were respectively evaluated using the Shapiro-Wilk and Levene’s tests. For compliant data, a 2-tailed independent samples *t* test, 1-way ANOVA with Tukey’s post hoc test, and 2-factor repeated-measures ANOVA were used. Noncompliant data were assessed via the Mann-Whitney *U* test, Kruskal-Wallis H test with Dunn’s post hoc test, 2-tailed Welch’s *t* test, or the Wilcoxon signed-rank test.

### Study approval.

All animal experiments strictly adhered to the IACUC at Nanfang Hospital, Southern Medical University. The animal experiment protocol received approval from the same committee. Clinical samples from Nanfang Hospital complied with national ethical regulations and were approved by the Nanfang Hospital Ethics Committee. All surgical and experimental procedures received approval from the Ethics Committee at Nanfang Hospital, Southern Medical University. All participating patients provided informed consent, and all specimens were coded to ensure patient confidentiality.

### Data availability.

The microarray data and scRNA-Seq data have been deposited in the GEO database under accession nos. GSE64287 and GSE126060, respectively. Supporting data for this study can be found within the article or supplemental materials, or they can be obtained from the corresponding author upon reasonable request. Values for all data points in graphs are reported in the Supporting Data Values file.

## Author contributions

TJ designed the experiments, analyzed the results, and wrote the manuscript. TJ, XA, and XX carried out most of the experiments. JZ assisted in collecting clinical samples. J Cai helped with data analysis. JF, WZ, Z Zheng, J Chu, and MH helped with some experiments. Z Zhang and LW supervised the experiments and proofread the manuscript.

## Supplementary Material

Supplemental data

Unedited blot and gel images

Supporting data values

## Version 1 11/26/2024

In-Press Preview

## Version 2 01/09/2025

Electronic publication
