# Supplementary material for: Mast cell activation by NGF drives the formation of trauma-induced heterotopic ossification
Source: JCI Insight. 2024 Nov 26;10(1):e179759. doi: 10.1172/jci.insight.179759 (PMC11721298; doi:10.1172/jci.insight.179759)
Supplement: Supplemental data [file jciinsight-10-179759-s247.pdf]

Supplementary Materials for

Mast cell activation by NGF drives the formation of trauma-induced heterotopic ossification

Tao Jiang[1,2], Xiang Ao[2], Xin Xiang[2], Jie Zhang[1], Jieyi Cai[3], Jiaming Fu[2], Wensheng Zhang[1], Zhenyu Zheng[1], Jun Chu[1], Minjun Huang[1], Zhongmin Zhang[2], Liang Wang[1]

1. Division of Spine Surgery, Department of Orthopedics, The Third Affiliated Hospital, Southern Medical University, Academy of Orthopedics, Guangdong Province, Guangzhou, China.
2. Division of Spine Surgery, Department of Orthopedics, Nanfang Hospital, Southern Medical University, Guangzhou, China.
3. Department of General Medicine, Zhujiang Hospital, Southern Medical University, Guangzhou, China.

Authorship note: TJ, XA, and XX contributed equally to this work.

Corresponding author: zzmzzc@smu.edu.cn (ZMZ); liang091@aliyun.com (LW)

The PDF file includes:

Supplemental Methods

Supplemental Figures 1-12

Supplemental Tables 1-7

Supplemental Methods

Protein homology analysis. Before using the human NT3 neutralizing antibody to neutralize mouse NT3, a preliminary analysis was conducted to determine whether the human NT3 neutralizing antibody (see Supplemental Table 7 for details) could neutralize mouse NT3. The FASTA sequence of human NT3 was first downloaded from NCBI (https://www.ncbi.nlm.nih.gov/). Next, the Protein BLAST module on the NCBI website was selected, and the FASTA sequence of human NT3 (see Supplemental Table 3 for details) was entered into the query box. The “Standard databases” were chosen, with the species database set to mouse. Finally, the homology between the two proteins was analyzed using the blastp (protein-protein BLAST) module (see Supplemental Table 4 for details).

In vitro treatment with anti-NT3 neutralizing antibody. TDSCs were cultured in 6-well plates (1×10^5 cells/well) under conditions of 37°C and 5% CO_2 until they reached 50% confluency, at which point they were transfected with *Ntf3*-lentiviral vectors (LV). Three days after transfection, when the TDSCs reached 90% confluency, the culture medium was replaced with a chondrogenic medium. To confirm the chondrogenic effects of NT3-TrkC signaling, TDSCs were cultured in a chondrogenic medium with 350 ng/ml neutralizing rabbit anti-human NT3 polyclonal antibody or 350 ng/ml control rabbit IgG monoclonal antibody (see Supplemental Table 7 for details) until the end of the 14 - day culture period. The medium was refreshed every 2-3 days. The control group received chondrogenic induction alone, and a standard medium culture group was used to assess the effectiveness of chondrogenic induction. TB staining and western

blotting were used to evaluate the experimental results described above (see Supplemental Figure 5).

In vivo treatment with LPS. To explore the synergistic effect of NGF and LPS in HO pathogenesis, LPS or rmNGF + LPS (see Supplemental Table 7) were administered to C57BL/6J mice 3 days before tenotomy. Specifically, LPS was dissolved in saline and intraperitoneally administered at 0.65 μg/g (1) body weight every other day. In another experimental group, mice were treated every two days with rmNGF at 4 ng/g (2, 3) following the intraperitoneal injection of LPS. All treatments were administered for 8 weeks following HO induction. Control mice received the same volume of saline as a control for intraperitoneal injections (see Supplemental Figure 3).

References

1. Salga M, et al. Bacterial Lipopolysaccharides Exacerbate Neurogenic Heterotopic Ossification Development. *J Bone Miner Res.* 2023.
2. Gao L, et al. Hearing Improvement in A/J Mice via the Mouse Nerve Growth Factor. *Clin Exp Otorhinolaryngol.* 2017;10(4):303-8.
3. Testa G, et al. The NGF(R100W) Mutation Specifically Impairs Nociception without Affecting Cognitive Performance in a Mouse Model of Hereditary Sensory and Autonomic Neuropathy Type V. *J Neurosci.* 2019;39(49):9702-15.

Supplemental Figures 1-12

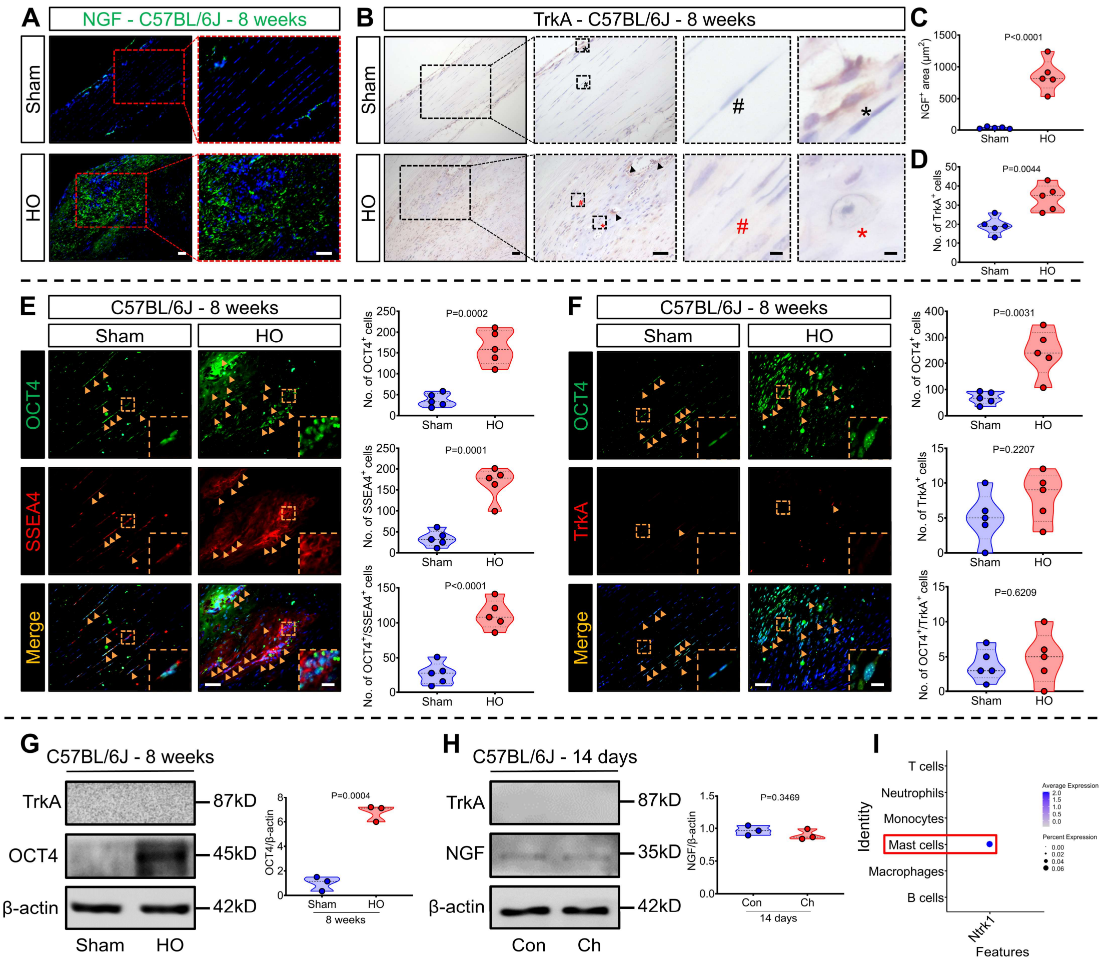

Supplemental Figure 1. TrkA is not expressed in TDSCs but specifically in mast cells during traumatic HO. (**A** and **C**) Representative IF staining (**A**) images of NGF (green) in C57BL/6J mice tendons of sham-operated and HO-induced groups at 8 weeks after tenotomy, with DAPI counterstaining (blue). (**C**) Positive NGF stained areas were quantified. Scale bar = 5 μm, n = 5 biological replicates. (**B** and **D**) Representative (**B**) IHC staining images and (**D**) quantification of TrkA of mouse Achilles tendon sections in the indicated groups 8 weeks. Black hash and asterisks represent tendon cells and subcutaneous mucosal cells in the sham-operated group, and red hash and asterisks represent tendon cells and osteocytes in the HO-induced group, respectively. The

number of positive cells was quantified, scale bar = 5 μm and 1μm (right 2 panels), n = 5 biological replicates. (**E** and **F**) Representative double-immunostaining images of OCT4$^+$ (green)/SSEA4$^+$ (red) and OCT4$^+$ (green)/TrkA$^+$ (red) cells in tendons, with DAPI counterstaining (blue). The number of positive cells was counted separately (right). Scale bar = 5 μm (left) and 1 μm (right), n = 5 biological replicates. (**G** and **H**) Western blotting and densitometric quantification (right) were performed to detect the expression of (**G**) TrkA and OCT4 in proteins extracted from injured tendon tissues and the expression of (**H**) TrkA and NGF in cellular proteins extracted from TDSCs, with β-actin serving as a loading control, n = 3 biological replicates. (**I**) The scRNA-seq analysis of dataset GSE126060 revealed the expression of *Ntrk1* in six types of immune cells (macrophage, neutrophil, mast cell, monocyte, T cell, and B cell) at all time points (days 0, 3, 7, 21) presented as bubble plots. Data were shown as mean ± SD, and compared with two-tailed unpaired Student's t-test (**C**, **D**, **E**, **F**, **G**, and **H**).

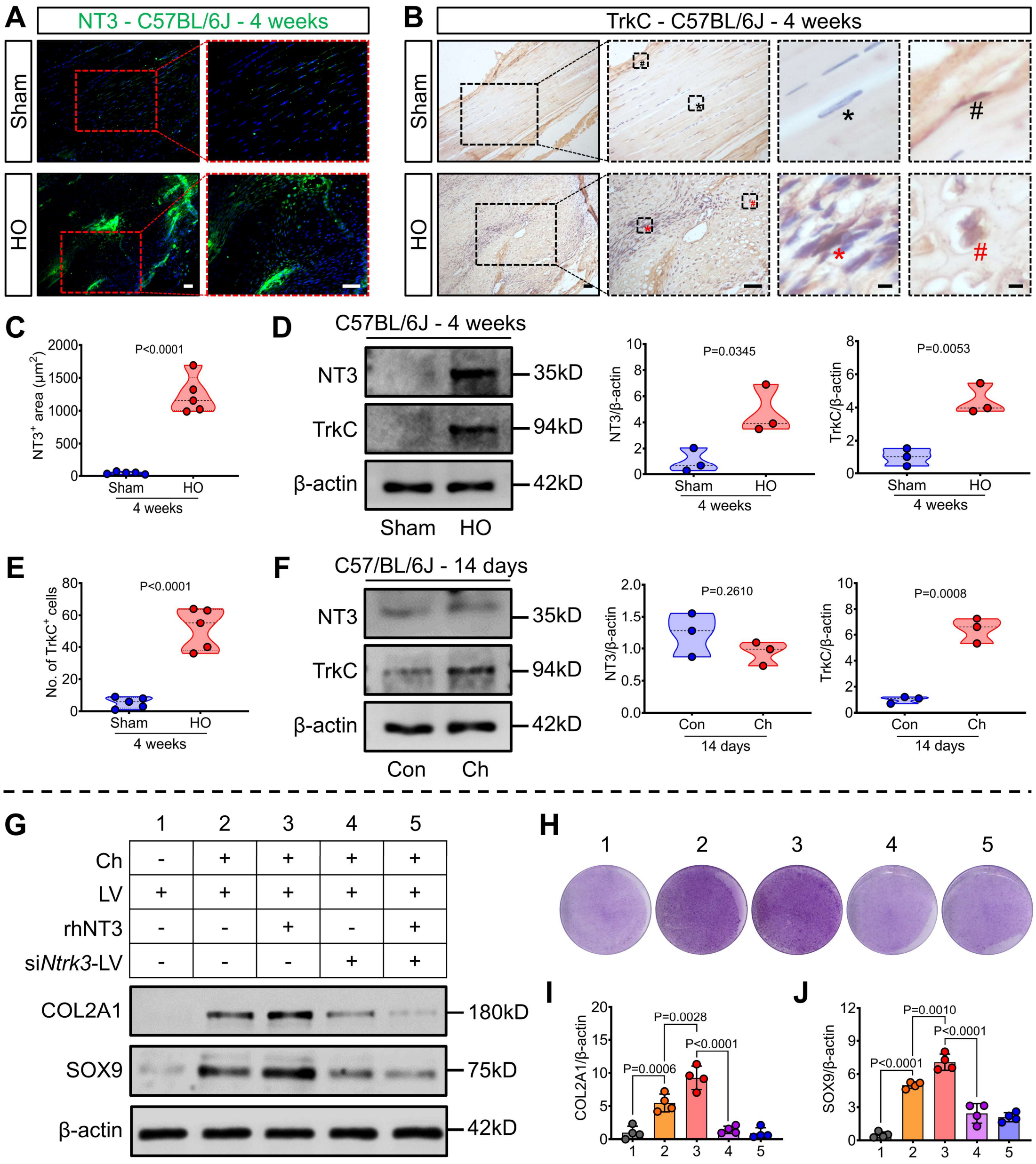

Supplemental Figure 2. NT3/TrkC is widely expressed in injured tendon tissue and markedly promotes chondrogenic differentiation of TDSCs. (**A** and **C**) Representative (**A**) IF staining images and (**C**) quantification of NT3 (green) in C57BL/6J mice tendons of sham-operated and HO-induced groups at 8 weeks after tenotomy, with DAPI counterstaining (blue). Positive NT3 stained areas were quantified. Scale bar = 5 μm, n = 5 biological replicates. (**B** and **E**) Representative (**B**) IHC staining images and (**E**) quantification of TrkC of mouse Achilles tendon sections

in the indicated groups. Black hash and asterisks represent tendon cells and subcutaneous mucosal cells in the sham-operated group, and red hash and asterisks represent tendon cells and chondrocytes in the HO-induced group, respectively. The number of positive cells was quantified, scale bar = 5 μm and 1μm (right 2 panels), n = 5 biological replicates. (**D** and **F**) Western blotting and densitometric quantification (right) were performed to detect the expression of NT3 and TrkC (**D**) in proteins extracted from injured tendon tissues or (**F**) in cellular proteins extracted from TDSCs, with β-actin serving as a loading control, n = 3 biological replicates. (**G**) Western blotting and (**H**) TB staining were performed on TDSCs challenged with rhNT3 (100 ng/ml), si*Ntrk3*-LV, or rhNT3 (100 ng/ml) + si*Ntrk3*-LV in chondrogenic culture for 14 days to assess chondrogenic differentiation, compared with the control (Con) group and chondrogenesis (Ch) alone group. (**I** and **J**) Densitometric quantification of western blotting was performed for (**I**) COL2A1 and (**J**) SOX9, with β-actin serving as a loading control, n = 3 biological replicates. Data were shown as mean ± SD, and compared with two-tailed unpaired Student's t-test (**C**-**F**) or one-way ANOVA with Tukey's multiple comparison test (**I** and **J**).

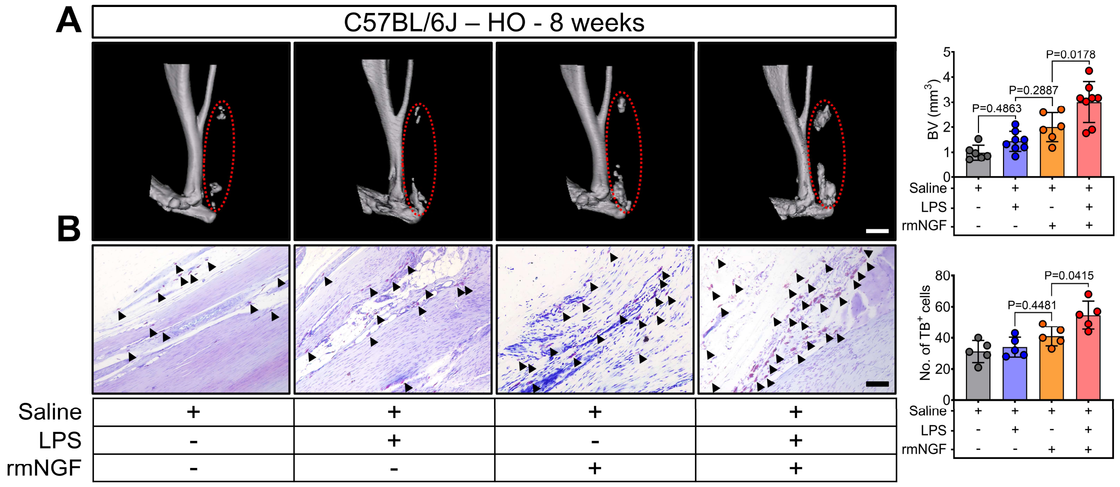

Supplemental Figure 3. NGF and LPS synergistically promote the progression of traumatic HO via mast cell activation in vivo. (**A**) Representative µCT 3D modeling images and quantification (right) of ectopic BV in mouse Achilles tendon sections from the indicated groups 8 weeks after tenotomy. Red dashed ovals represent ectopic bones. Scale bar = 2 mm, n = at least 6 biological replicates. (**B**) Representative TB staining images and quantification (right) of the mast cells in mouse Achilles tendon sections treated with either rmNGF, LPS, or rmNGF+LPS groups after tenotomy. The total number of mast cells was counted. Black arrows indicate mast cells. Scale bar = 5 µm, n = 5 biological replicates. Data were shown as mean ± SD, and compared with one-way ANOVA with Tukey's multiple comparison test.

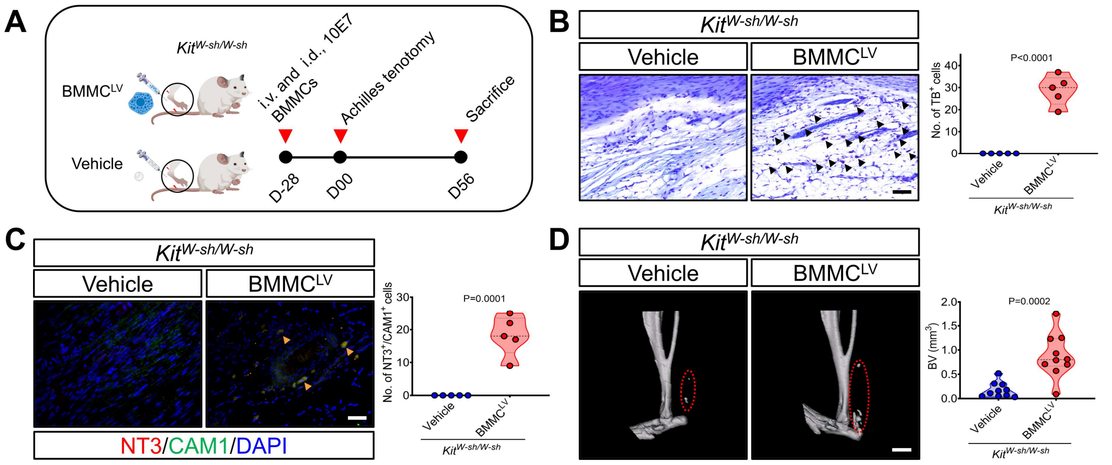

Supplemental Figure 4. Mast cell reconstitution resumes traumatic HO in *Kit$^{W-sh/W-sh}$* mice. (**A**) Scheme of the experimental protocol of i.v. and i.d. transfer of mast cells into *Kit$^{W-sh/W-sh}$* mice for the generation of HO mouse model. (**B**) TB staining and quantification (right) of cells with positive staining of tendon tissue sections of *Kit$^{W-sh/W-sh}$* mice with vehicle (PBS) or BMMCs by LV (Lentiviral vectors) transfected, n = 5 biological replicates. (**C**) Representative double-immunostaining images and quantification (right) of NT3$^+$ (red)/CAM1$^+$ (green) cells in the tendon sections from the indicated groups, with DAPI counterstaining (blue). The number of co-localized positive cells was counted. Yellow arrows indicate mast cells. Scale bar = 5 μm, n = 5 biological replicates. (**D**) μCT 3D reconstruction, along with quantification (right) of ectopic BV and BA, was performed. The red ellipse dashed box represents the reconstruction image of the ectopic bone. Scale bar = 2 mm, n = 10 biological replicates. Data were shown as mean ± SD, and compared with two-tailed unpaired Student's t-test.

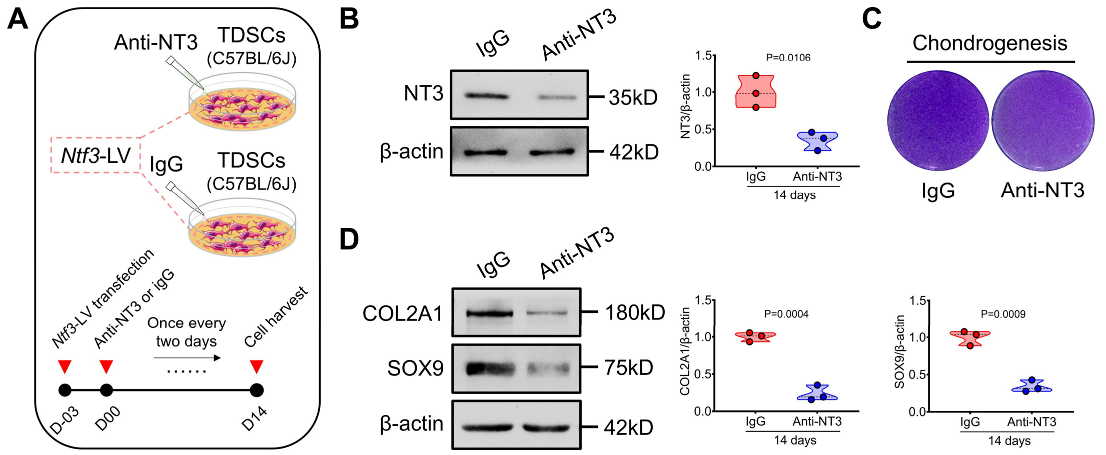

Supplemental Figure 5. Neutralization of NT3 attenuates chondrogenic differentiation of TDSCs in vitro. (**A**) Schematic representation of the in vitro neutralization assay. (**B**) Representative western blotting results and their densitometric quantification (right) showing the efficiency of NT3 neutralization, with β-actin serving as the loading control, n = 3 biological replicates. (**C** and **D**) Representative (**C**) TB staining results, and (**D**) western blotting results and their densitometric quantification (right) of COL2A1 and SOX9 in TDSCs after treatment with anti-NT3 or vehicle (IgG) for 14 days of chondrogenic induction. β-actin was used as the loading control, n = 3 biological replicates. Data were shown as mean ± SD, and compared with two-tailed unpaired Student's t-test.

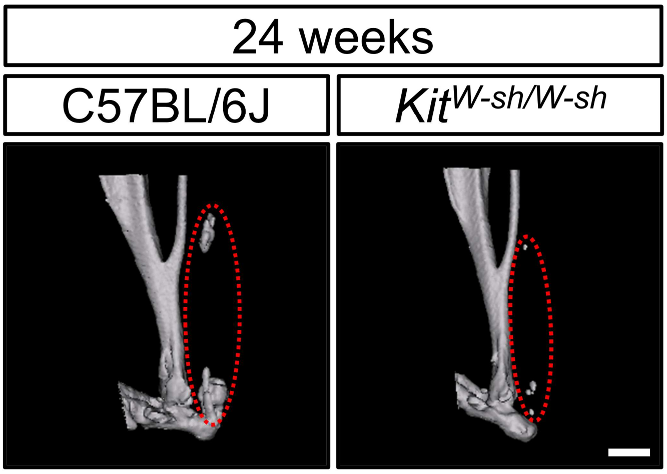

Supplemental Figure 6. Development of traumatic HO in C57BL/6J and *Kit*$^{W\text{-}sh/W\text{-}sh}$ mice 24 weeks after tenotomy, related to Figure 1A. Representative μCT 3D modeling images of Achilles tendon (sagittal view) of mice in indicated group 24 weeks (late osseous phase) after tenotomy. Red dashed ovals represent ectopic bones. Scale bar = 2 mm.

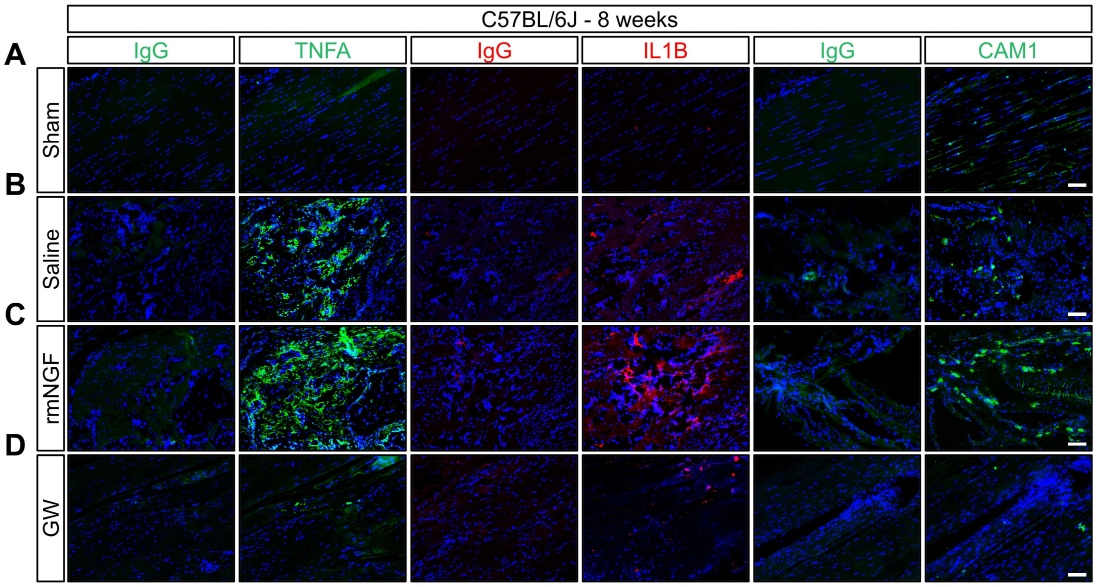

Supplemental Figure 7. Expression of TNFA, IL1B, and CAM1 in injured tendons 8 weeks after tenotomy, related to Figure 5, A, B, and D. (**A**) Isotype control (IgG) and TNFA, IL1B, and CAM1 IF staining results of injured tendon sections in C57BL/6J mice 8 weeks after Sham operation. Scale bar = 5 µm. (**B**) Isotype control (IgG) and TNFA, IL1B, and CAM1 IF staining in injured tendon sections of C57BL/6J mice after 8 weeks of Saline i.p. injection following Achilles tenotomy. Scale bar = 5 µm. (**C**) Isotype control (IgG) and TNFA, IL1B, and CAM1 IF staining in injured tendon sections of C57BL/6J mice after 8 weeks of rmNGF i.p. injection following Achilles tenotomy. Scale bar = 5 µm. (**D**) Isotype control (IgG) and TNFA, IL-1B, and CAM1 IF staining in injured tendon sections of C57BL/6J mice after 8 weeks of GW i.p. injection following Achilles tenotomy. Scale bar = 5 µm.

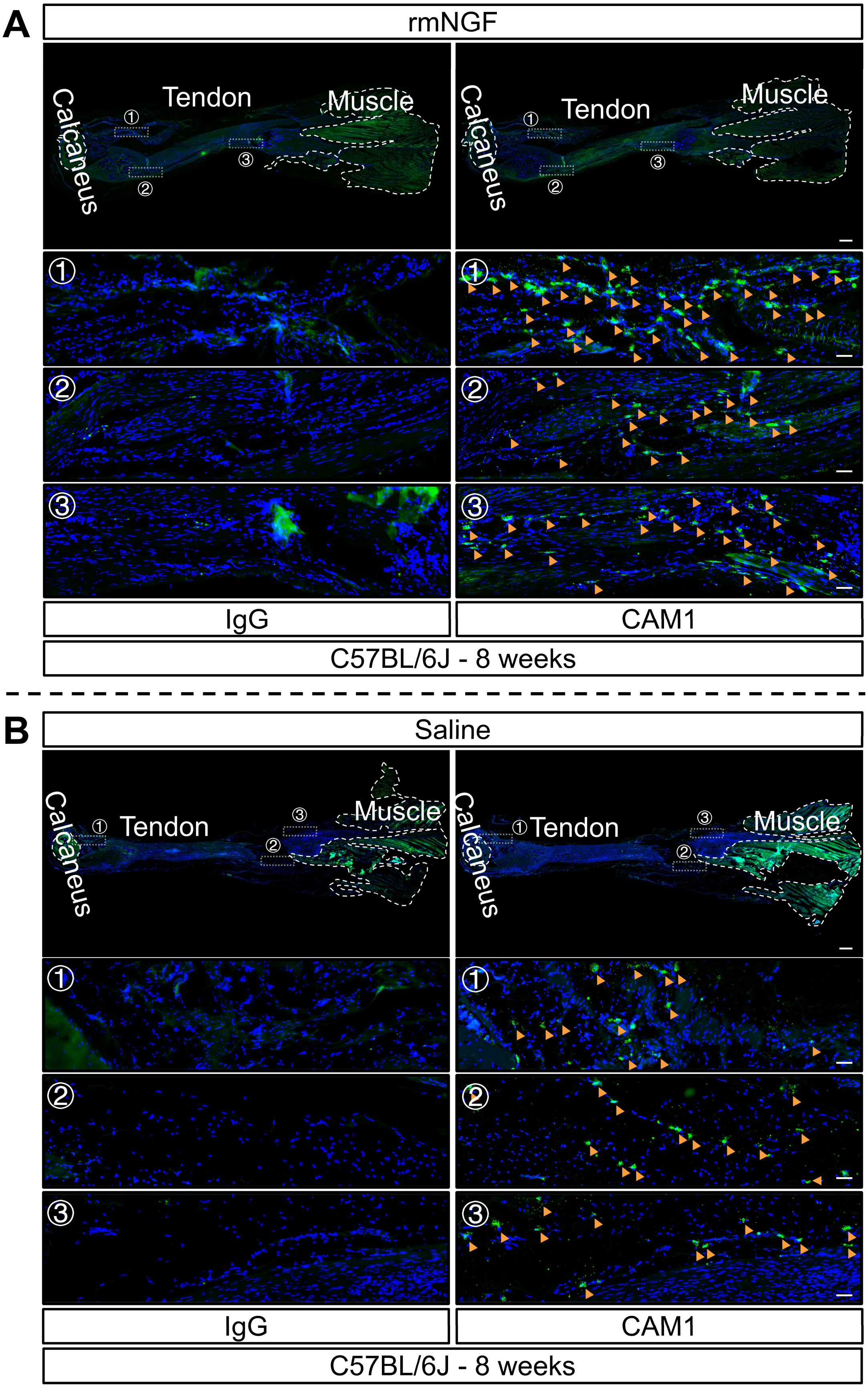

Supplemental Figure 8. Expression of CAM1 in the whole injured tendon sections

of the indicated group 8 weeks after tenotomy, related to Figure 5B. (**A**) Tile scan results of IF images for CAM1 (right) and isotype control (IgG, left) in injured tendon sections of C57BL/6J mice after 8 weeks of rmNGF i.p. injection following Achilles tenotomy. Panels ①-③ indicate different regions within the tendon. Scale bar = 500 μm (top panel) and 50 μm (①-③ panels). (**B**) Tile scan results of IF images for CAM1 (right) and isotype control (IgG, left) in injured tendon sections of C57BL/6J mice after 8 weeks of Saline i.p. injection following Achilles tenotomy. Panels ①-③ indicate different regions within the tendon. Scale bar = 500 μm (top panel) and 50 μm (①-③ panels).

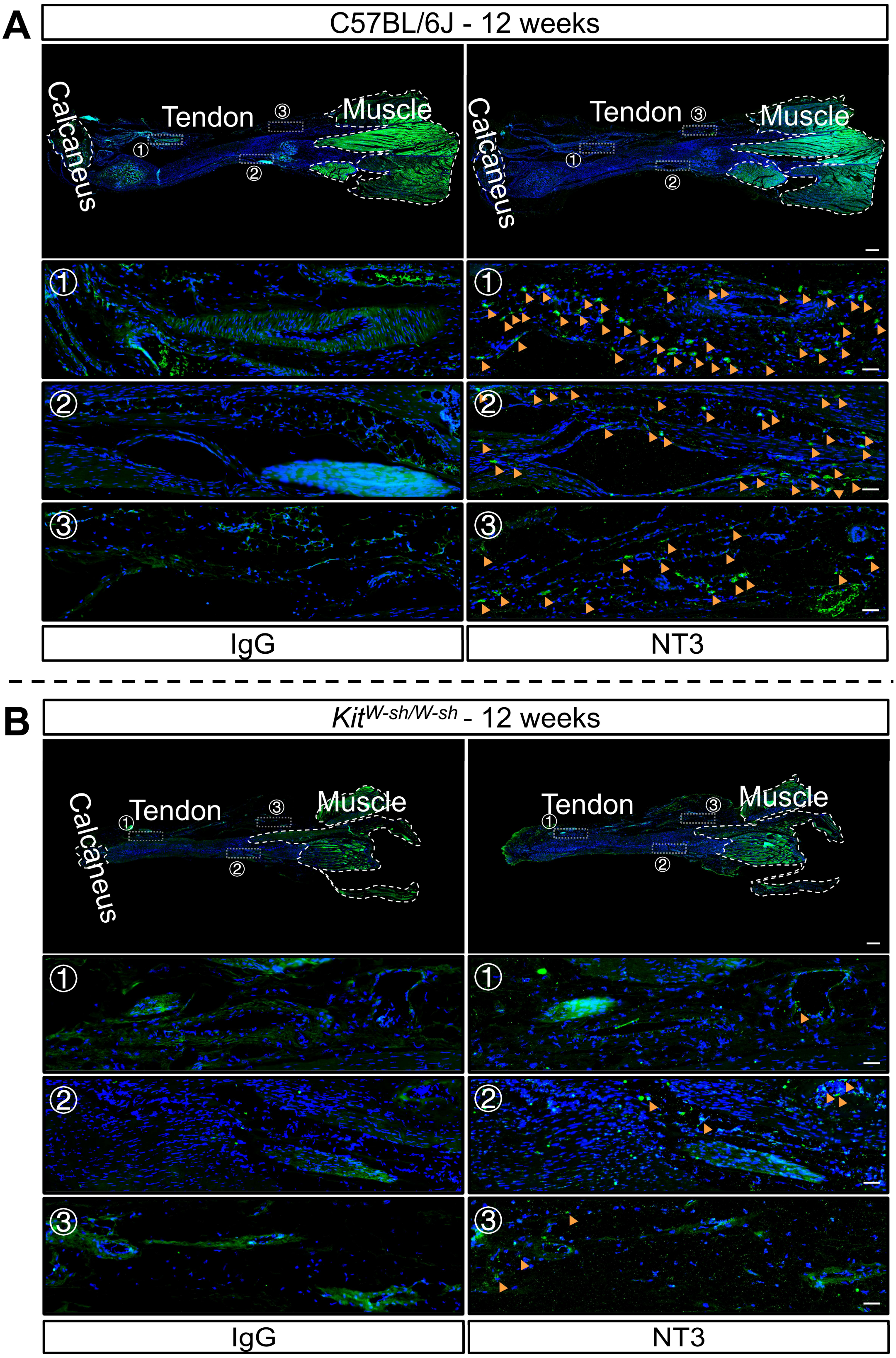

Supplemental Figure 9. Expression of NT3 in the whole injured tendon sections of the indicated group 12 weeks after tenotomy, related to Figure 6D. (**A**) Tile scan

results of IF images for NT3 (right) and isotype control (IgG, left) in injured tendon sections of C57BL/6J mice 12 weeks after Achilles tenotomy. Panels ①-③ indicate different regions within the tendon. Scale bar = 500 μm (top panel) and 50 μm (①-③ panels). (**B**) Tile scan results of IF images for NT3 (right) and isotype control (IgG, left) in injured tendon sections of *Kit*$^{W\text{-}sh/W\text{-}sh}$ mice 12 weeks after Achilles tenotomy. Panels ①-③ indicate different regions within the tendon. Scale bar = 500 μm (top panel) and 50 μm (①-③ panels).

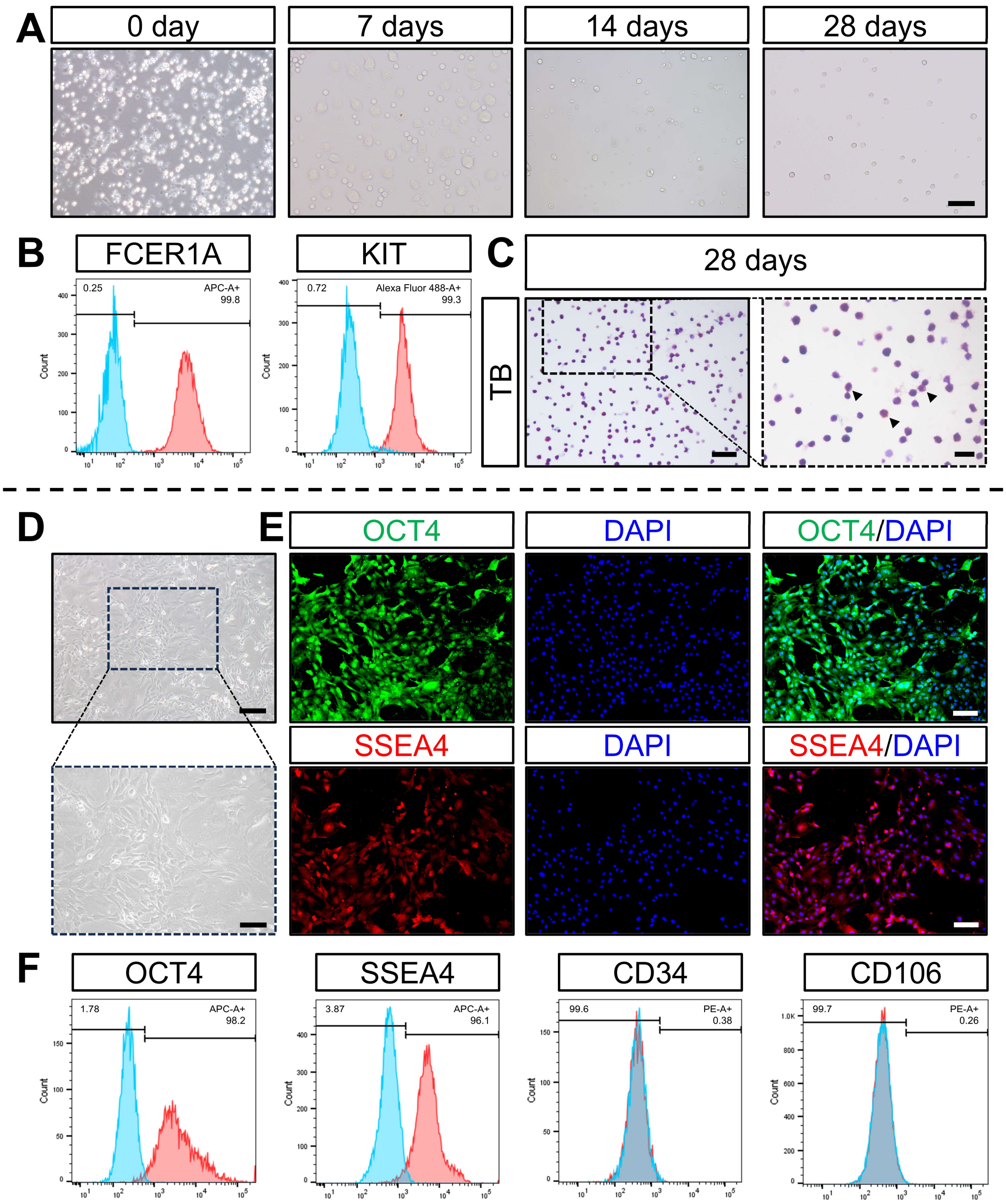

Supplemental Figure 10. Morphological and purity characterization of BMMCs (Bone marrow-derived mast cells) and TDSCs (Tendon-derived stem cells). (**A**) Cell morphology at different induced culturing periods under light microscopy. (**B**) Detection of mast cell-specific marker proteins FCER1A and KIT expression in BMMCs after 28 days of induction using flow cytometry. (**C**) Toluidine blue staining analysis of BMMCs after 28 days of induction. (**D**) Light microscopic cell morphology

of TDSCs. (**E**) The expression of TDSC-specific marker proteins OCT4 and SSEA4 was detected by cellular immunofluorescence. (**F**) Flow cytometric characterization of stemness markers (positive marker: OCT4, SSEA4, negative marker: CD34, CD106) in TDSCs.

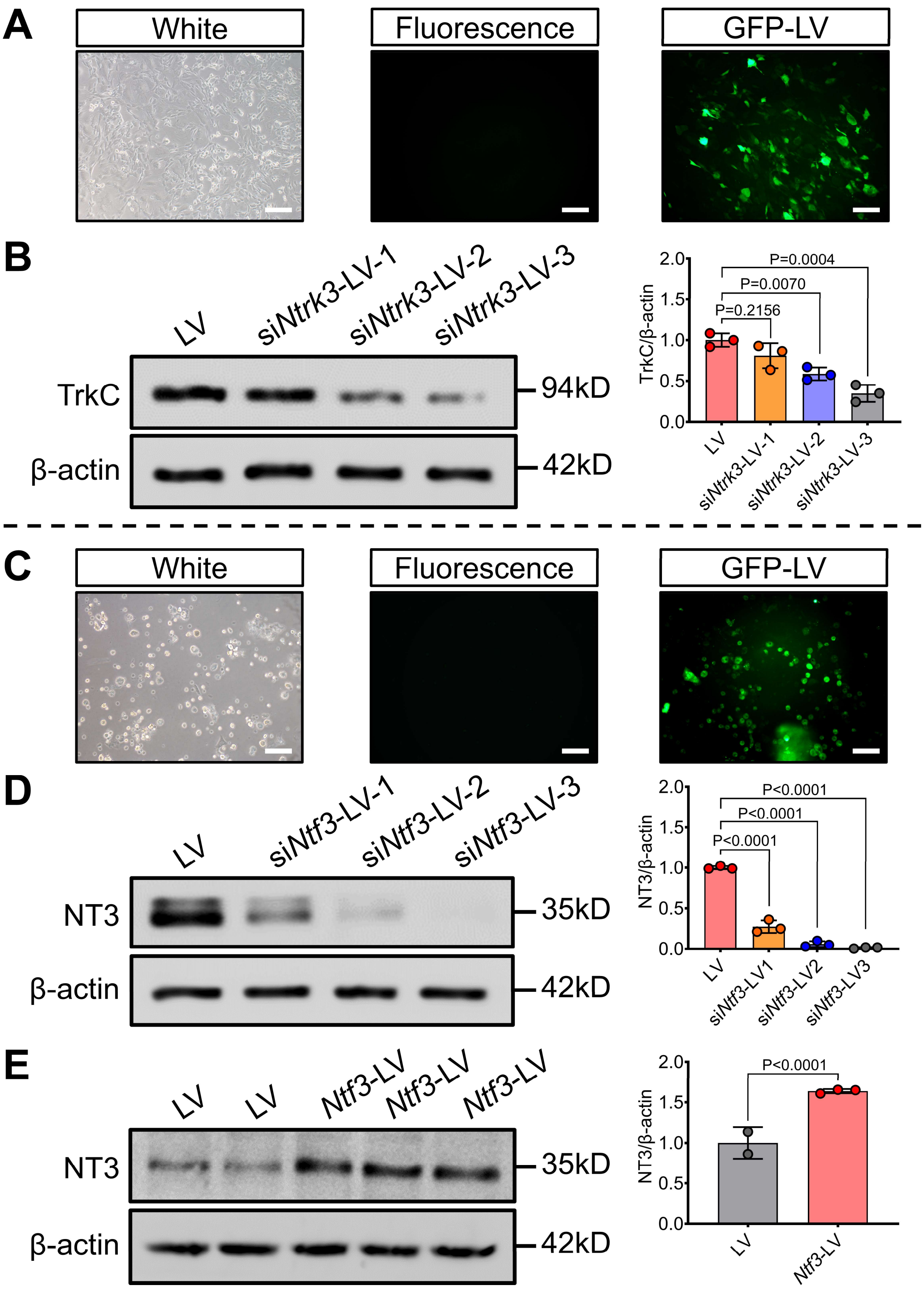

Supplemental Figure 11. Validation of lentiviral transfection and knockdown efficiency of TrkC in mouse TDSCs, as well as knockdown or overexpression efficiency of NT3 in mouse BMMCs. (**A**) Lentiviral vectors (LV) containing Green fluorescent protein (GFP) or si*Ntrk3* were transfected into TDSCs. The efficiency of

lentiviral transfection was visualized using inverted fluorescence microscopy, including both white and fluorescence light. Scale bar = 100μm. (**B**) The knockdown effectiveness of TrkC in TDSCs was assessed by western blotting and its densitometric quantification (right), n = 3 biological replicates per group. (**C**) Lentiviral vectors containing GFP-LV, si*Ntf3*-LV, and *Ntf3*-LV were transfected into BMMCs, both for 24 hours. The efficiency of lentiviral transfection was visualized using inverted fluorescence microscopy, including both white and fluorescence light. Scale bar = 100μm. (**D** and **E**) The knockdown effectiveness (**D**) and overexpression (**E**) of NT3 in BMMCs were assessed by western blotting and its densitometric quantification (right), n = 2 - 3 biological replicates per group. Data were shown as mean ± SD, and compared with one-way ANOVA with Tukey's multiple comparison test.

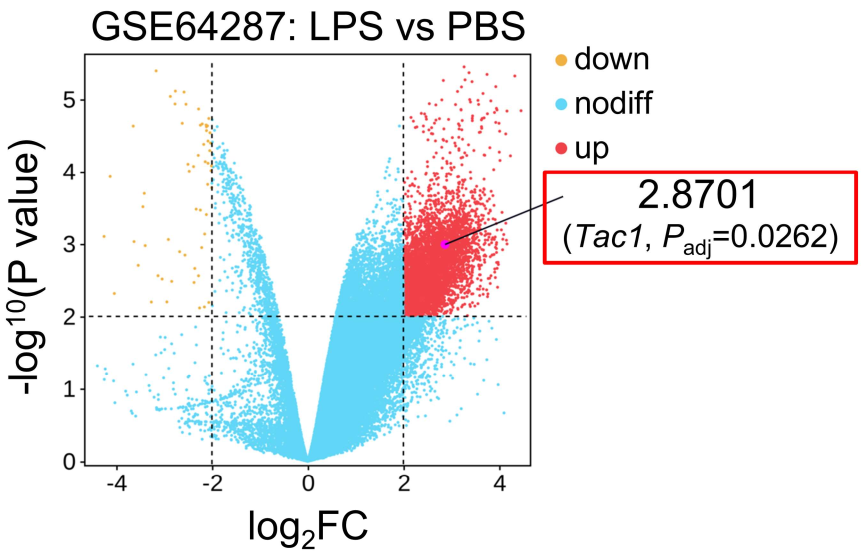

Supplemental Figure 12. *Tac1* (the gene encoding substance P) expression in BMMCs treated with LPS. Volcano plot of upregulated (red) and downregulated (yellow) DEGs in BMMCs treated with LPS (100ng/ml) for 1 hour from the dataset GSE64287. Expression of the *Tac1* (red box) is markedly increased ($P_{adj} < 0.05$, $log_2FC > 2$).

Supplemental Tables 1-7

Supplemental Table 1. The small interfering RNA (siRNA) sequences of NT3 and TrkC

**Name**	**Sequence 5'→3'**	
si*Ntf3*-1	Sense	GGCGCUGGAUACGAAUAGA
	Antisense	UCUAUUCGUAUCCAGCGCC
si*Ntf3*-2	Sense	CACAGAGCUACUACGGCAA
	Antisense	UUGCCGUAGUAGCUCUGUG
si*Ntf3*-3	Sense	CGCUAUGCAGAACAUAAGA
	Antisense	UCUUAUGUUCUGCAUAGCG
Control	Sense	UUGUACUACACAAAAGUACUG
	Antisense	GUACUUUUGUGUAGUACAGUU
si*Ntrk3*-1	Sense	CAGAGAACGUGGUGGGCAUTT
	Antisense	AUGCCCACCACGUUCUCUGTT
si*Ntrk3*-2	Sense	GCACAGAUUUCUUUGACUUTT
	Antisense	AAGUCAAAGAAAUCUGUGCT
si*Ntrk3*-3	Sense	ACACAGUGGUCAUUGGCAUTT
	Antisense	AUGCCAAUGACCACUGUGUTT
Control	Sense	UUCUCCGAACGUGUCACGUTT
	Antisense	ACGUGACACGUUCGGAGAATT

Supplemental Table 2. The overexpression sequence of NT3

GCCACCATGTGGCAGCCCCCGTCAGCCAGGATAATGATGAGGCAGATCTT
ACAGGTGAACAAGGTGATGTCCATCTTGTTTATGTGATATTTCTTGCTTA
TCTCCGTGGCATCCAAGGCAACAGCATGGATCAAAGGAGTTTGCCGGAA
GACTCTCTCAATTCCCTCATCATCAAGCTGATCCAGGCGGATATCTTGAAA
AACAAGCTTTCCAAACAGATGGTGGATGTTAAGGAAAATTACCAGAGCA
CCCTGCCCAAAGCAGAGGCACCCAGGGAACCAGAGCAGGGAGAGGCCA
CCAGGTCAGAGTTCCAGCCAATGATTGCAACGGACACAGAGCTACTACG
GCAACAGAGACGCTACAATTCGCCCCGGGTCCTGCTGAGTGACAGCACC
CCTTTGGAGCCCCCTCCCTTATACCTAATGGAGGATTATGTGGGCAACCC
GGTGGTAGCCAATAGAACCTCACCACGGAGGAAACGCTATGCAGAACAT
AAGAGTCACCGAGGAGAGTACTCAGTGTGTGACAGTGAGAGCCTGTGG
GTGACCGACAAGTCCTCAGCCATTGACATTCGGGGACACCAGGTCACAG
TGCTGGGGGAGATCAAAACCGGTAACTCTCCTGTGAAACAATATTTTTAT
GAAACGAGATGTAAAGAAGCCAGGCCGGTCAAAAACGGTTGCAGGGGG
ATTGATGACAAACACTGGAACTCTCAGTGCAAAACTTCGCAAACCTATGT
CCGAGCACTGACTTCAGAAAACAACAAACTCGTAGGCTGGCGCTGGATA
CGAATAGACACTTCCTGTGTGTGTGCCTTGTCGAGAAAAATTGGAAGAA
CATGA

Supplemental Table 3. The FASTA sequence of human NT3 protein

MSILFYVIFLAYLRGIQGNNMDQRSLPEDSLNSLIIKLIQADILKNKLSKQMV
DVKENYQSTLPKAEAPREPERGGPAKSAFQPVIAMDTELLRQQRRYNSPRV
LLSDSTPLEPPPLYLMEDYVGSPVVANRTSRRKRYAEHKSHRGEYSVCDSES
LWVTDKSSAIDIRGHQVTVLGEIKTGNSPVKQYFYETRCKEARPVKNGCRG
IDDKHWNSQCKTSQTYVRALTSENNKLVGWRWIRIDTSCVCALSRKIGRT

Supplemental Table 4. Comparison of homology results between human NT3 and mouse NT3 proteins

Description	Scientific name	Total Score	Query Cover	E value	Per. Ident	Acc. Len	Accession
NT3 isoform a precursor	M. musculus	513	100%	0.0	95.74%	271	NP_001157506.1
NT3 isoform b preproprotein	M. musculus	512	100%	0.0	95.74%	258	NP_001397057.1
NT3 isoform c precursor	M. musculus	511	100%	0.0	95.74%	278	NP_001397058.1
NT3, isoform CRA_c	M. musculus	197	40%	3e-64	91.43%	105	EDK99838.1
NT3, isoform CRA_a	M. musculus	97.4	18%	1e-25	97.87%	47	EDK99836.1
β-NGF isoform B precursor	M. musculus	183	99%	1e-56	41.22%	241	NP_001106168.1
β-NGF isoform A	M. musculus	182	99%	2e-55	41.00%	307	NP_038637.1

Supplemental Table 5. The experimental mouse lines used

No.	Mouse strain	Source
1#	C57BL/6J	Laboratory Animal Management Center of Southern Medical University
2#	*Kit*$^{W\text{-}sh/W\text{-}sh}$	Jackson Laboratory, Stock #012861

Supplemental Table 6. Human trauma and HO sample demographic data

Case No.	Trauma Period (Mean, days)	Age (Mean, years)	Gender (M/F)	Location	Causes
1#	0	38.33	M	Achilles tendon	Accident
2#	0	38.33	M	Achilles tendon	Accident
3#	0	38.33	F	Achilles tendon	Accident
4#	0	38.33	F	Achilles tendon	Accident
5#	7	38.33	M	Achilles tendon	Sports injury
6#	7	38.33	M	Achilles tendon	Sports injury
7#	7	38.33	F	Achilles tendon	Sports injury
8#	7	38.33	F	Achilles tendon	Sports injury
9#	365~	38.33	M	PLL	OPLL
10#	365~	38.33	M	PLL	OPLL
11#	365~	38.33	F	PLL	OPLL
12#	365~	38.33	F	PLL	OPLL

M: Male; F: Female

Supplemental Table 7. Reagents used

Name	Vendor	Catalog No.	Concentration	Use
Anti-NT3	Proteintech	18084-1-AP	1:1000/100/100	WB, IHC, IF
Anti-TrkC	Proteintech	11999-1-AP	1:1000/100	WB, IHC
Anti-NGF	HUABIO	ET1606-29	1:1000/100/100	WB, IHC, IF
Anti-TrkA	HUABIO	ET1608-44	1:1000/100	WB, IHC
Anti-TrkA^{Tyr490}	Affinity	AF2429	1:1000	WB
Anti-LPS	Sigma	SAB4200882	1:5000	WB
Anti-LPS	Feiyuebio	FY-AB33259	1:40	IP
Anti-TLR4	Proteintech	66350-1-Ig	1:1000	WB
Anti-β-actin	HUABIO	M1210-2	1:10000	WB
Anti-SOX9	Abcam	ab185966	1:1000/200	WB, IF
Anti-COL2A1	Servicebio	GB11021	1:1000/100	WB, IF
Anti-OCT4	Abcam	ab200834	1:2000/50	WB, IF
Goat Anti-Mouse IgG	ABclonal	AS003	1:10000/200	WB, IHC
Goat Anti-Rabbit IgG	ABclonal	AS014	1:10000/200	WB, IHC
Anti-CAM1	Affinity	DF12290	1:200	IHC
Anti-RUNX2	Abclonal	A2851	1:50	IF
Anti-OCN	Abclonal	A6205	1:50	IF
Anti-IL1B	Abclonal	A19635	1:50	IF
Anti-TNFA	Abclonal	A0277	1:50	IF
Anti-rabbit IgG	Abclonal	AC042	1:50-200	IHC, IF

Anti-mouse IgG	Abclonal	AC011	1:50-200	IHC, IF
proteinase K	Beyotime	ST533	1:100	IHC
Anti-FCER1A	Proteintech	10980-1-AP	1:100	IF
Anti-KIT	Bioss	bs-10005R	1:100	IF
Anti-SSEA4	Abcam	ab16287	1:50	IF
Anti-CD16/32	BioLegend	101301	1:50	FCM
Anti-FCER1A	Invitrogen	17-5898-80	1:200	FCM
Anti-KIT	Invitrogen	11-1171-81	1:100	FCM
Anti-CAM1	Proteintech	18189-1-AP	1:100	IF
Anti-rabbit IgG	Proteintech	30000-0-AP	1:50-200	IHC, IF
Anti-SSEA4	BioLegend	330417	1:20	FCM
Anti-OCT4	BioLegend	653703	1:20	FCM
Anti-CD34	BioLegend	128609	1:80	FCM
Anti-CD106	BioLegend	105703	1:50	FCM
Goat anti-Rabbit IgG-488	Invitrogen	A11008	1:400	IF
Goat anti-Rabbit IgG-594	Invitrogen	A32740	1:400	IF
Goat anti-Mouse IgG-488	Invitrogen	A32723	1:400	IF
Goat anti-Mouse IgG-594	Invitrogen	A11005	1:400	IF
rmHis-TrkA	MCE	HY-P76116	5μM	IP
rmFc-TrkA	MCE	HY-P76115	5μM	IP
Anti-His tag	Beyotime	AF2873	1:500	IP
Anti-Fc tag	Abcam	ab97265	1:10000	IP

PMSF	Beyotime	ST506	1:100	WB
Type I collagenase	Sigma	C0130	1mg/ml	In vitro
Rabbit anti-human NT3	Origene	TA388898	350ng/ml	In vitro
Isotype Control IgG	Origene	TA385792	350ng/ml	In vitro
GW	MCE	HY-18314	1μM	In vitro
GW	MCE	HY-18314	10ug/g/day	In vivo
rmNGF	Peprotech	450-34	100ng/ml	In vitro
rmNGF	Peprotech	450-34	4ng/g/day	In vivo
LPS	Sigma	L4391-1MG	100ng/ml	In vitro
LPS	Sigma	L4391-1MG	0.65μg/g	In vivo
rhNT3	Peprotech	450-03	100ng/ml	In vitro
RD	MCE	HY-11109	1μM	In vitro
rmIL-3	Peprotech	213-13	10ng/ml	In vitro
rmSCF	Peprotech	250-03	10ng/ml	In vitro
rhTGFB3	Peprotech	100-36E	10ng/ml	In vitro
Ascorbic acid	Sigma	A4403	50μg/ml	In vitro
Dexamethasone	Sigma	D4902	10nM	In vitro
ITS-premix	Sigma	I3146	1:100	In vitro

WB: Western blot; IP: Immunoprecipitation; FCM: Flow cytometry; IF: Immunofluorescence; IHC: Immunohistochemistry
