# Supplementary material for: Mast cell activation by NGF drives the formation of trauma-induced heterotopic ossification
Source: JCI Insight. 2024 Nov 26;10(1):e179759. doi: 10.1172/jci.insight.179759 (PMC11721298; doi:10.1172/jci.insight.179759)

NGF and TrkA full unedited gel for Figure 3C

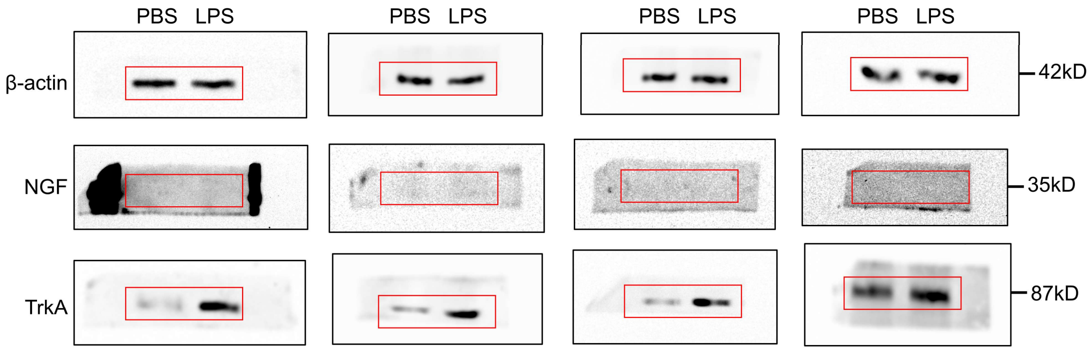

SOX9 and COL2A1 full unedited gel for Figure 4K

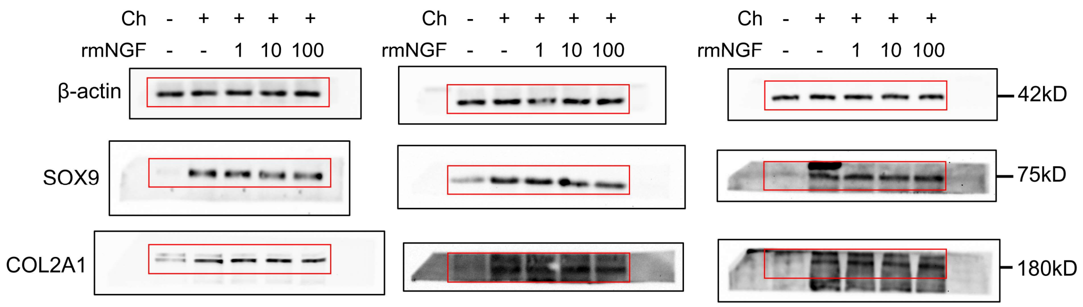

SOX9 and COL2A1 full unedited gel for Figure 4L

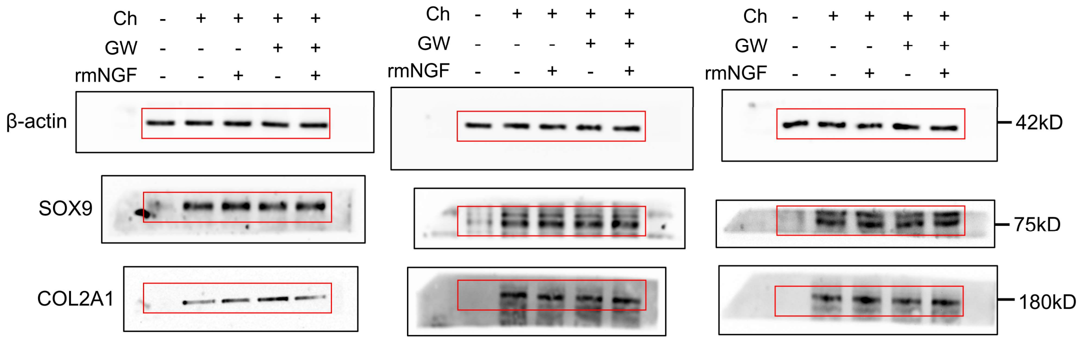

NT3 and TrkC full unedited gel for Figure 6B

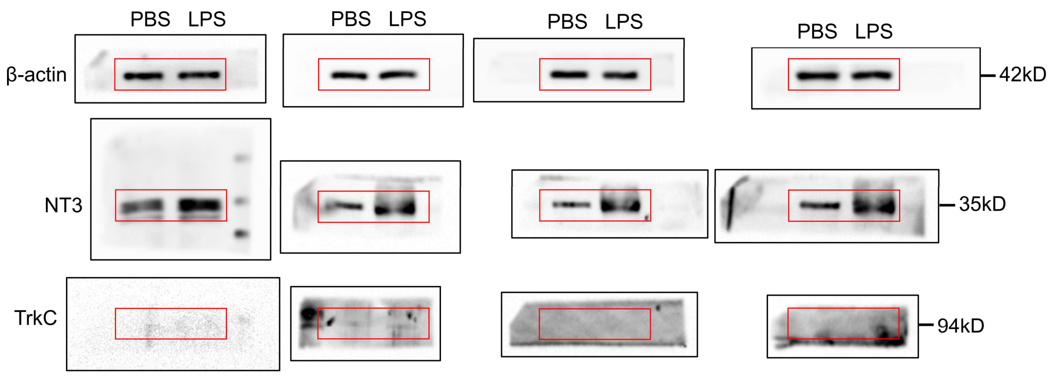

NT3 full unedited gel for Figure 7A

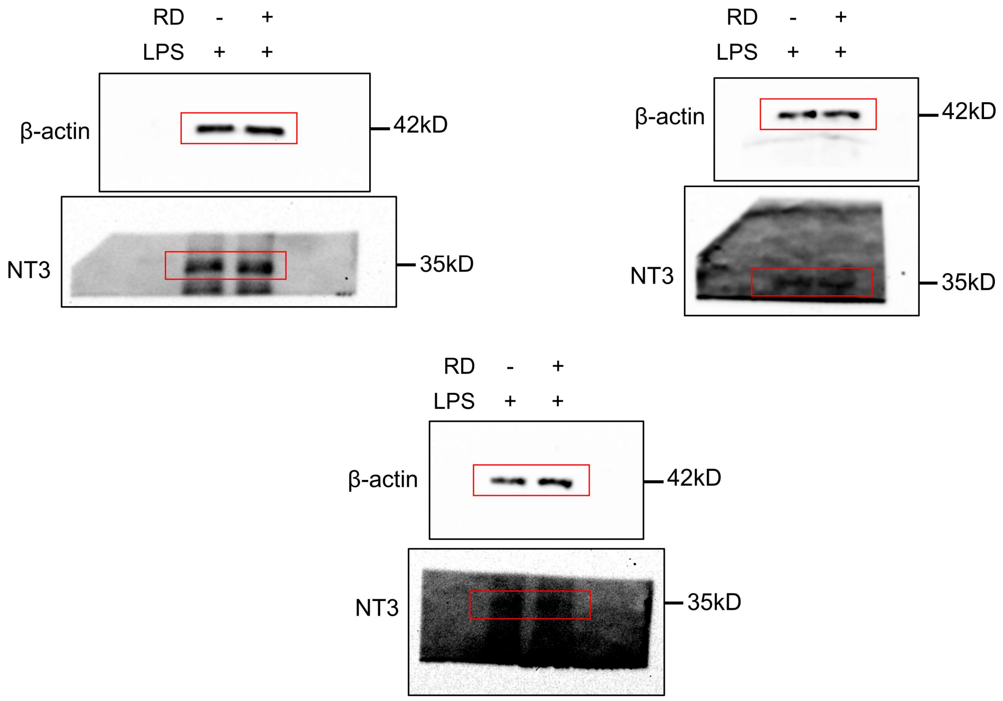

TLR4 full unedited gel for Figure 7C

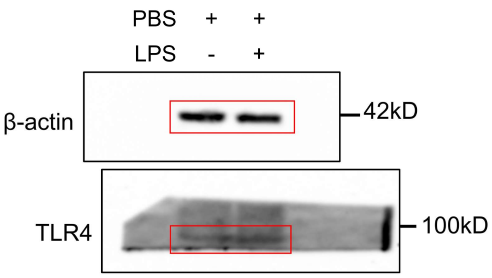

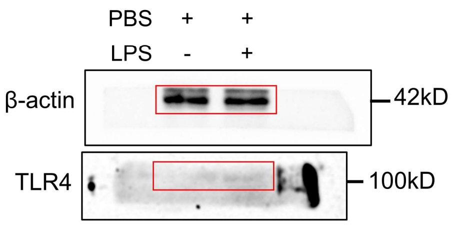

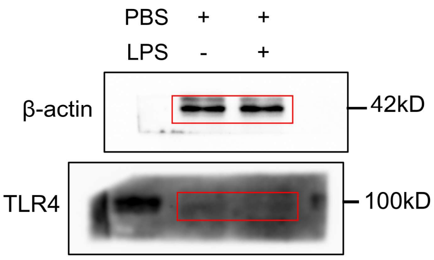

IP full unedited gel for Figure 7D

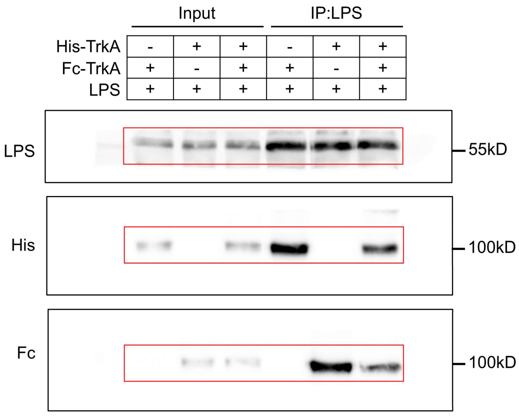

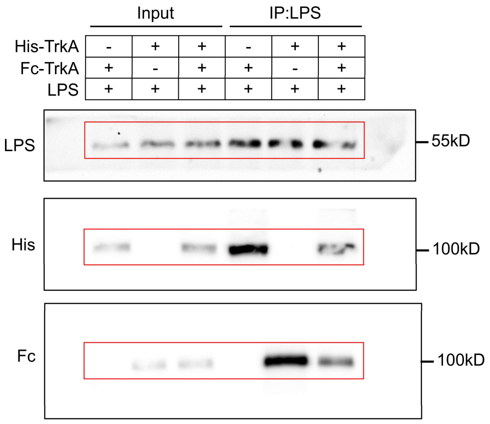

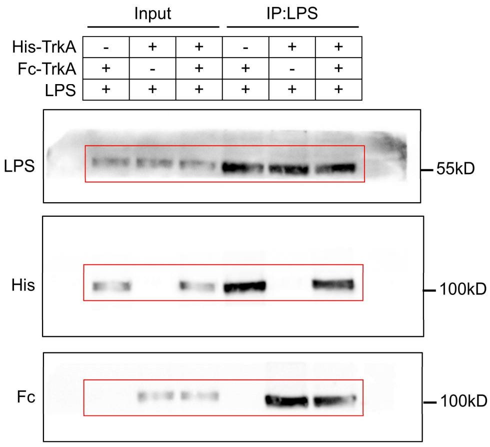

TrkA^{Tyr490} full unedited gel for Figure 7J

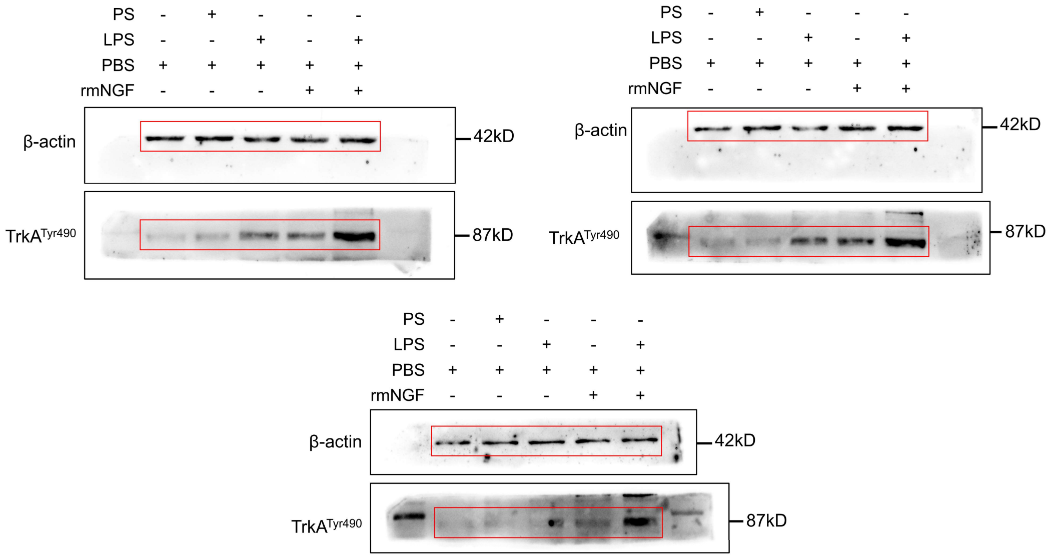

NT3 full unedited gel for Figure 7K

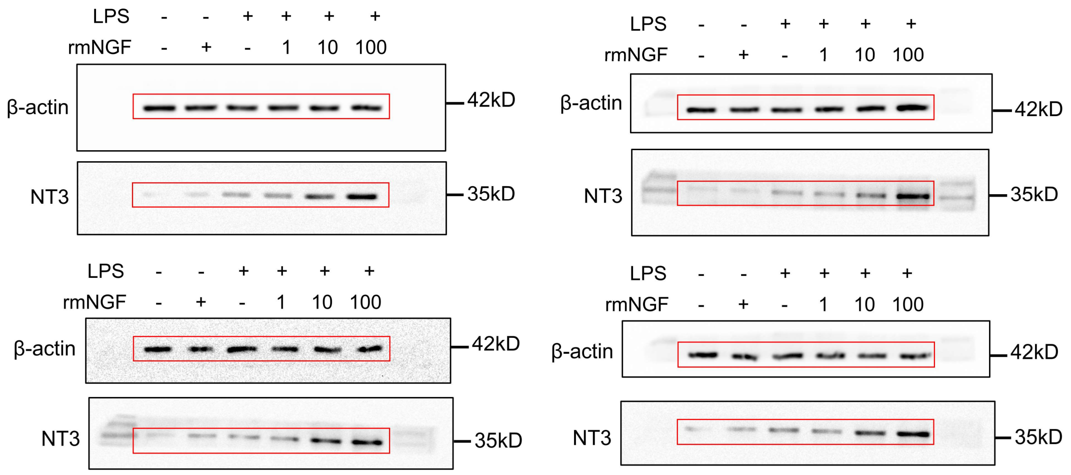

NT3 full unedited gel for Figure 7L

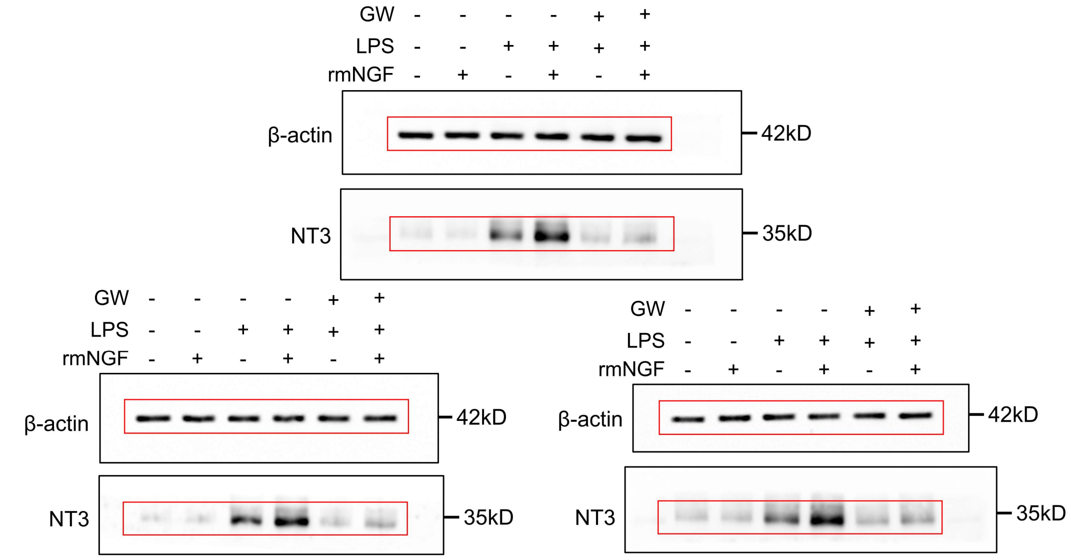

SOX9 and COL2A1 full unedited gel for Figure 8C

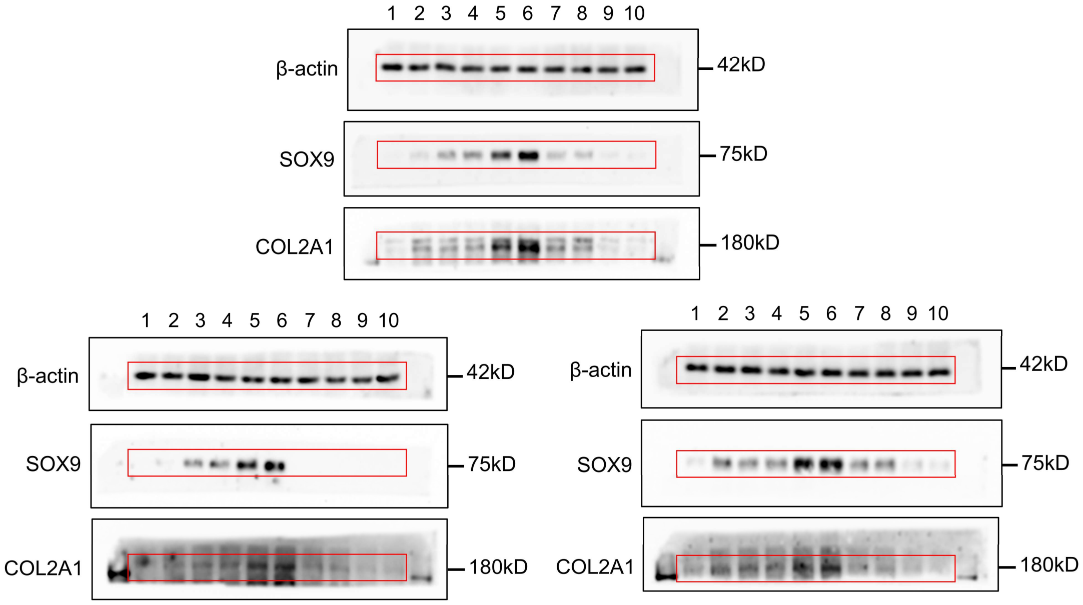

TrkA and OCT4 full unedited gel for Supplemental Figure 1G

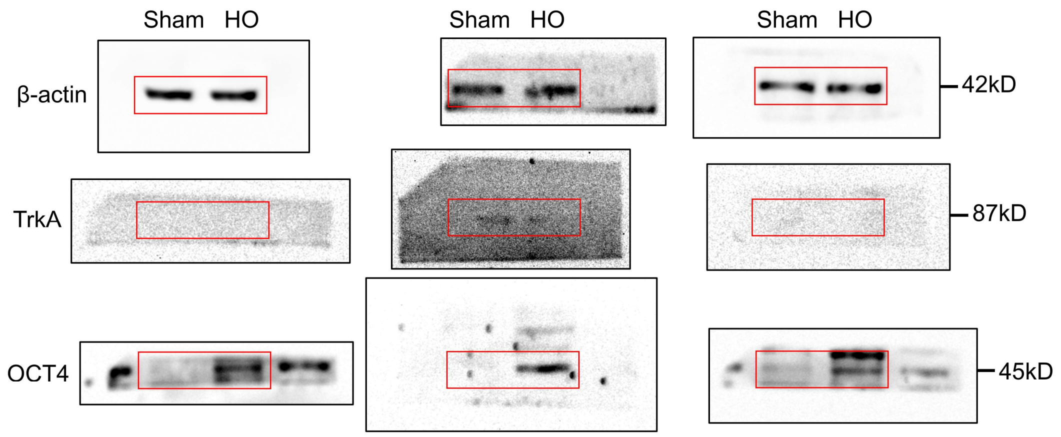

NGF and TrkA full unedited gel for Supplemental Figure 1H

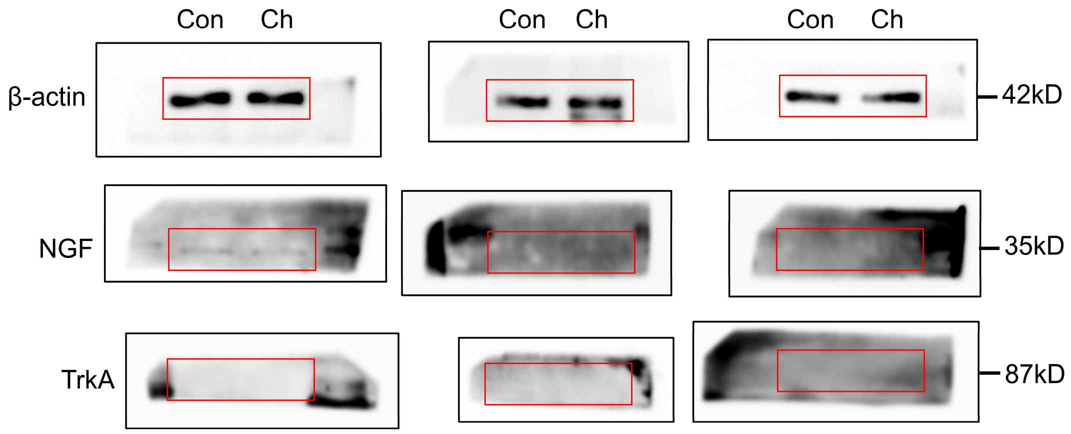

NT3 and TrkC full unedited gel for Supplemental Figure 2D

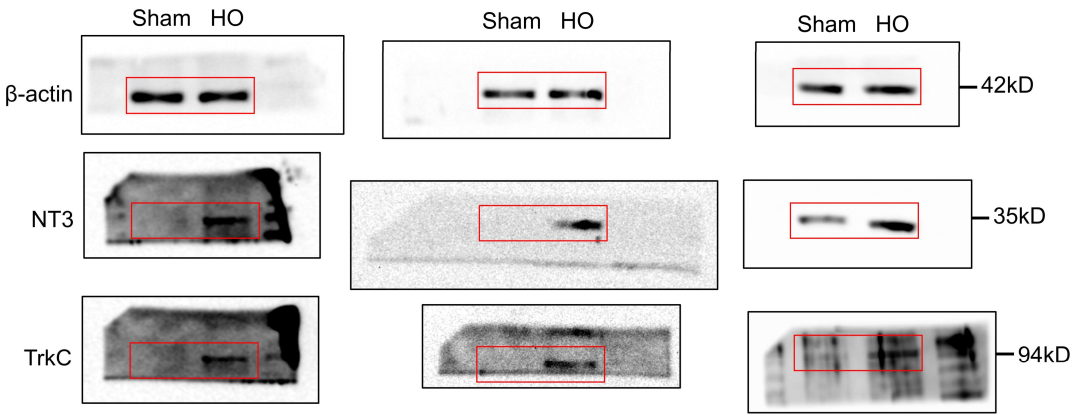

NT3 and TrkC full unedited gel for Supplemental Figure 2F

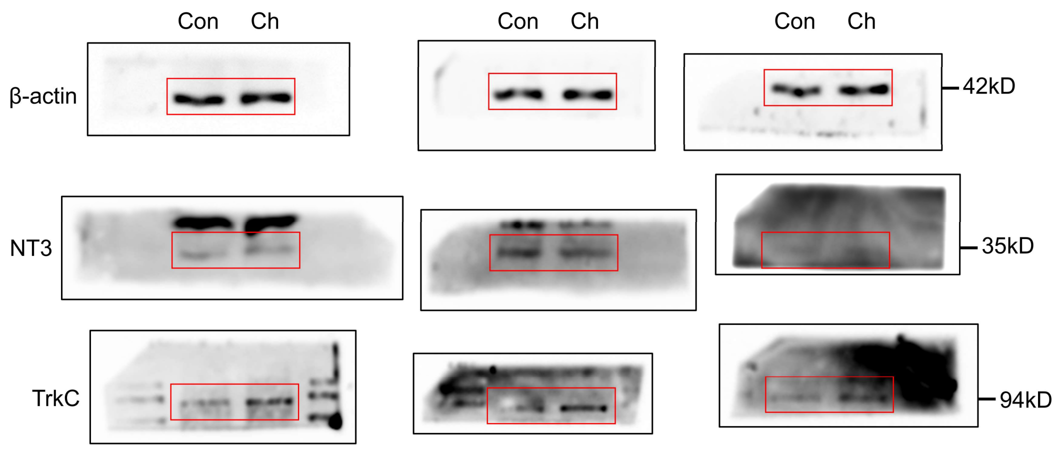

SOX9 and COL2A1 full unedited gel for Supplemental Figure 2G

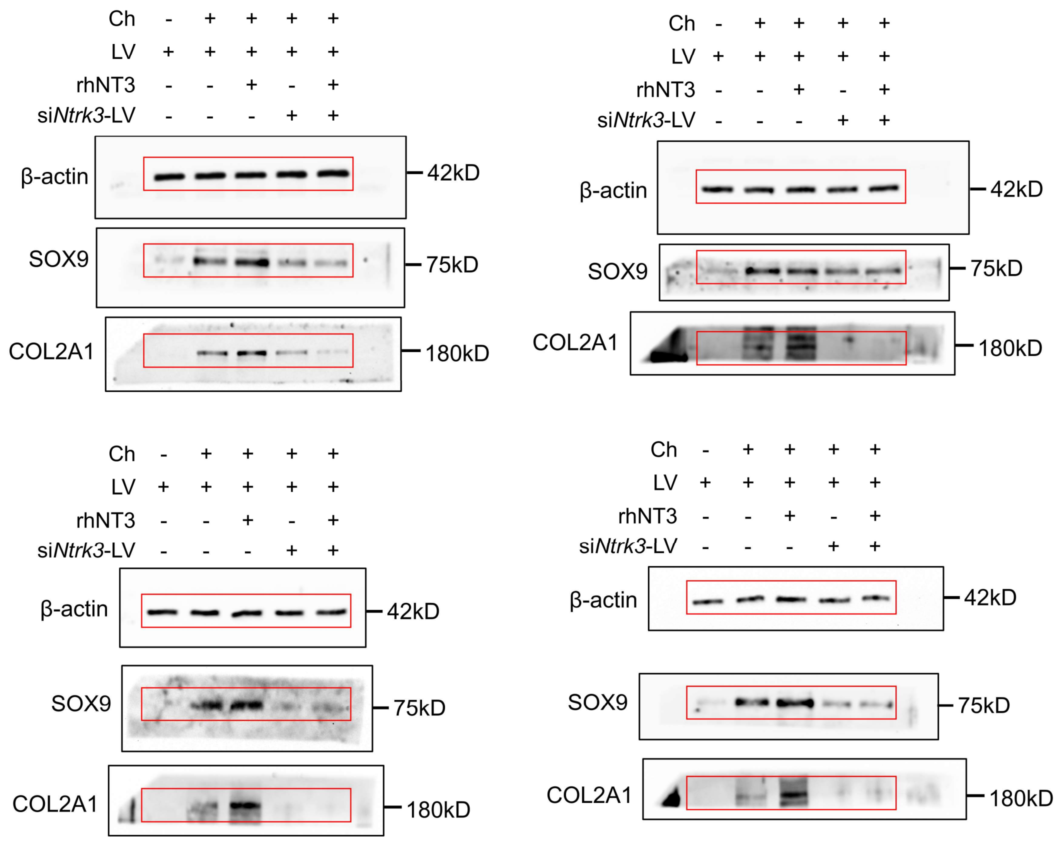

NT3 full unedited gel for Supplemental Figure 5B

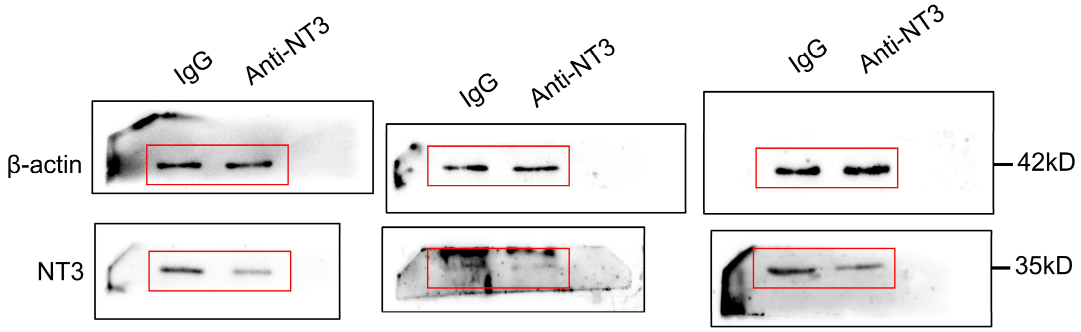

SOX9 and COL2A1 full unedited gel for Supplemental Figure 5D

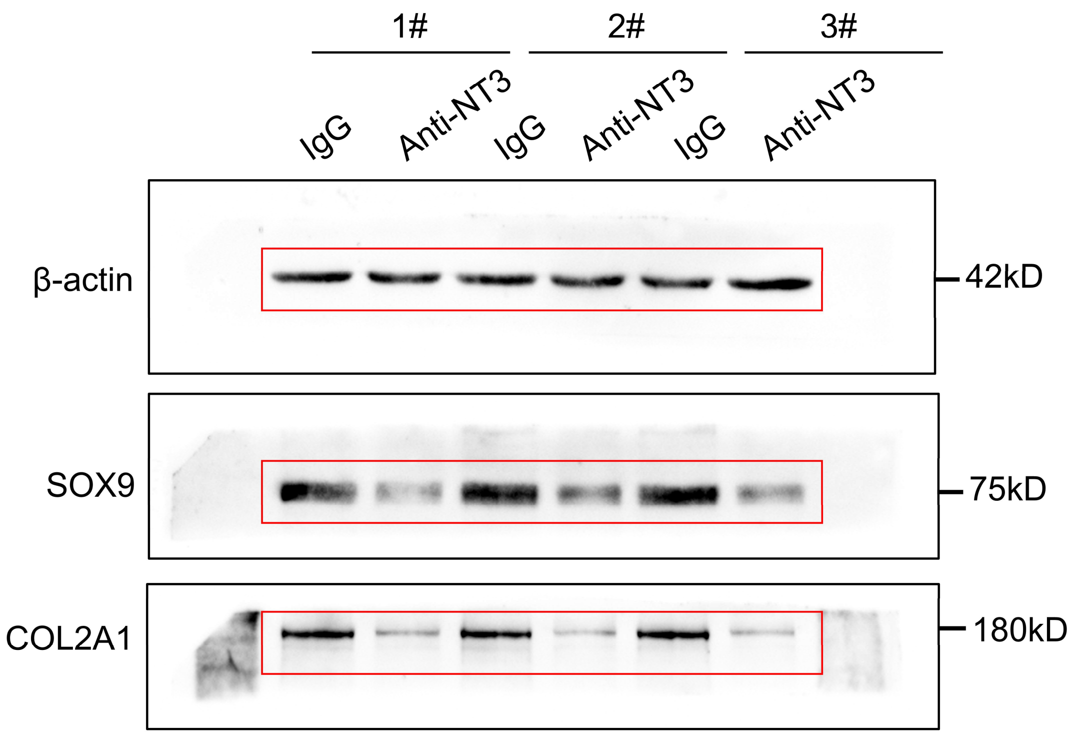

TrkC full unedited gel for Supplemental Figure 12B

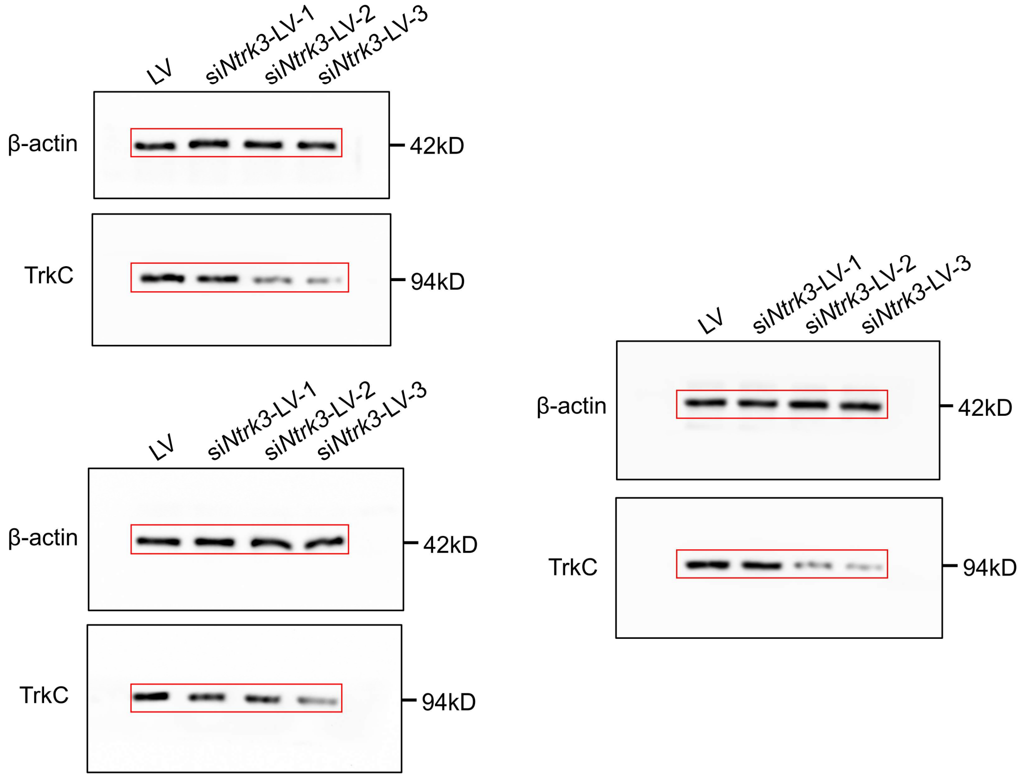

NT3 full unedited gel for Supplemental Figure 12D

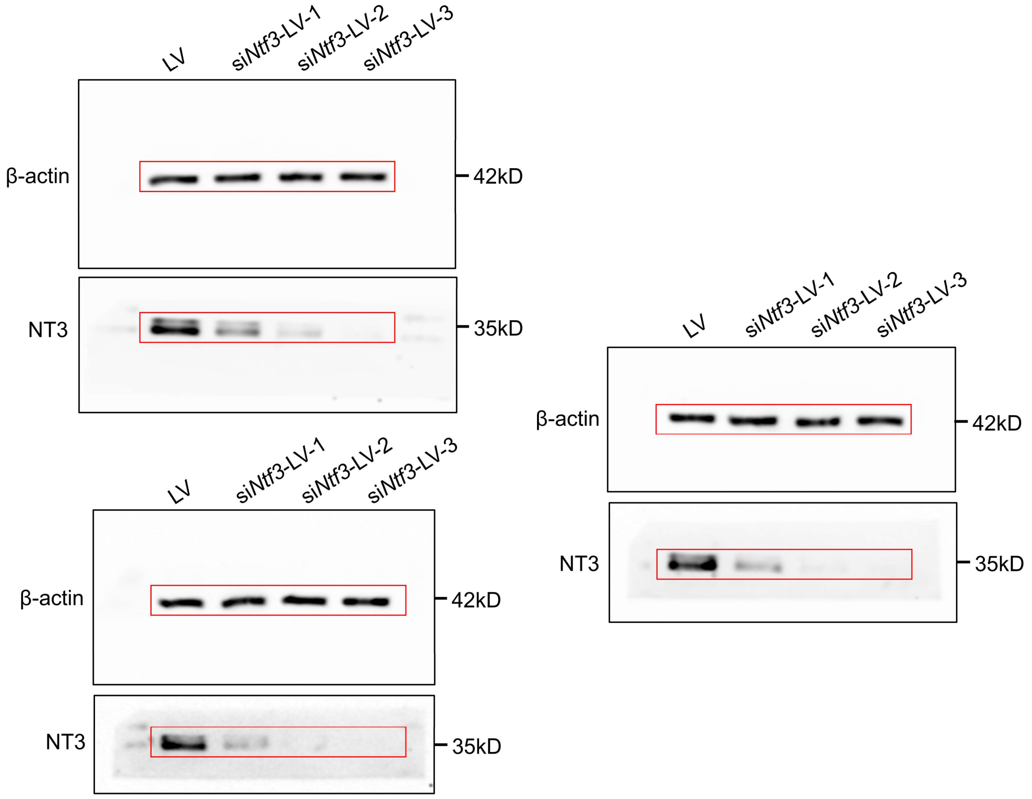

Ntf3 full unedited gel for Supplemental Figure 12E

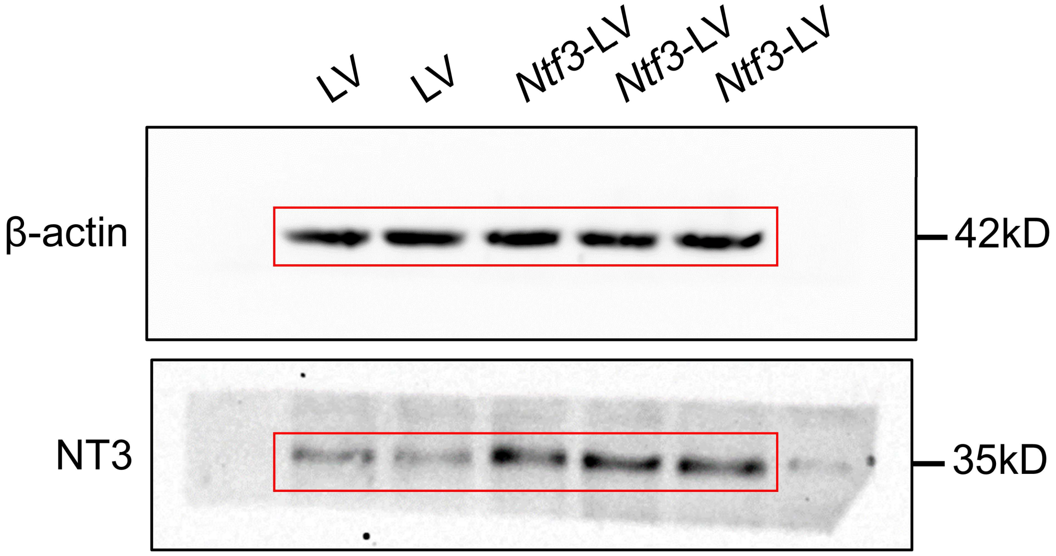

Supplement: Unedited blot and gel images [file jciinsight-10-179759-s248.pdf]
